# Chemical Composition of the Red Sea Green Algae *Ulva lactuca*: Isolation and In Silico Studies of New Anti-COVID-19 Ceramides

**DOI:** 10.3390/metabo11120816

**Published:** 2021-11-29

**Authors:** Enas E. Eltamany, Sameh S. Elhady, Marwa S. Goda, Omar M. Aly, Eman S. Habib, Amany K. Ibrahim, Hashim A. Hassanean, Usama Ramadan Abdelmohsen, Martin K. Safo, Safwat A. Ahmed

**Affiliations:** 1 Department of Pharmacognosy, Faculty of Pharmacy, Suez Canal University, Ismailia 41522, Egypt; enastamany@gmail.com (E.E.E.); marwa_saeed@pharm.suez.edu.eg (M.S.G.); emy_197@hotmail.com (E.S.H.); am_kamal66@yahoo.com (A.K.I.); hashem_omar@pharm.suez.edu.eg (H.A.H.); 2 Department of Natural Products, Faculty of Pharmacy, King Abdulaziz University, Jeddah 21589, Saudi Arabia; ssahmed@kau.edu.sa; 3 Department of Medicinal Chemistry, Faculty of Pharmacy, Minia University, 61519 Minia, Egypt; omarsokkar@yahoo.com; 4 Department of Pharmacognosy, Faculty of Pharmacy, Minia University, Minia 61519, Egypt; usama.ramadan@mu.edu.eg; 5 Department of Pharmacognosy, Faculty of Pharmacy, Deraya University, New Minia 61111, Egypt; 6 Department of Medicinal Chemistry, and Institute for Structural Biology, Drug Discovery and Development, School of Pharmacy, Virginia Commonwealth University, Richmond, VA 23219, USA; msafo@vcu.edu

**Keywords:** COVID-19, *Ulva lactuca*, ceramides, SARS-CoV-2, hACE2, M^pro^, metabolic profiling

## Abstract

Coronavirus disease 2019 (COVID-19) is the disease caused by the virus SARS-CoV-2 responsible for the ongoing pandemic which has claimed the lives of millions of people. This has prompted the scientific research community to act to find treatments against the SARS-CoV-2 virus that include safe antiviral medicinal compounds. The edible green algae *U.* *lactuca*. is known to exhibit diverse biological activities such as anti-influenza virus, anti-Japanese encephalitis virus, immunomodulatory, anticoagulant, antioxidant and antibacterial activities. Herein, four new ceramides in addition to two known ones were isolated from *Ulva* *lactuca*. The isolated ceramides, including **Cer-1, Cer-2, Cer-3, Cer-4, Cer-5** and **Cer-6** showed promising antiviral activity against SARS-CoV-2 when investigated using in silico approaches by preventing its attachment to human cells and/or inhibiting its viral replication. **Cer-4** and **Cer-5** were the most effective in inhibiting the human angiotensin converting enzyme (hACE)–spike protein complex which is essential for the virus to enter the human host. In addition to this, **Cer-4** also showed an inhibition of the SARS-CoV-2 protease (M^pro^) that is responsible for its viral replication and transcription. In this study, we also used liquid chromatography coupled to electrospray ionization high-resolution mass spectroscopy (LC-ESI-HRMS) to identify several metabolites of *U.* *lactuca*, including metabolites such as fatty acids, their glyceride derivatives, terpenoids, sterols and oxysterols from the organic extract. Some of these metabolites also possessed promising antiviral activity, as previously reported.

## 1. Introduction

Coronaviruses (CoVs) are positive RNA genome viruses belonging to the Coronaviridae family of the Nidovirales order, which is divided into four genera (A, B, C, D). Severe acute respiratory syndrome coronavirus 2 (SARS-CoV-2) belongs to the B genus [1]. In March 2020, the World Health Organization (WHO) declared that the coronavirus disease 2019 (COVID-19) outbreak was a pandemic. A novel coronavirus is a new strain that has not been previously identified in humans. CoVs have at least four structural proteins: namely spike protein, cover protein, membrane protein and nucleocapsid protein. The spike protein promotes host attachment and viral cell membrane fusion during virus infection [1]. Nowadays, scientific research is oriented towards the discovery of safe therapeutic agents that would cure COVID-19 or reduce its prevalence. Potential anti-coronavirus treatments can be divided into two main categories: one operating on the human immune system or human cells, and the other on the coronavirus itself [2]. Viruses often bind to receptor proteins on the surfaces of cells to enter human cells, for example, by linking with the human angiotensin-converting enzyme 2 (hACE2) receptor [3,4,5]. Protein–protein docking showed that SARS-CoV-2 spike proteins have a strong affinity for hACE2 [6]. Therefore, determining safe and natural compounds that inhibit COVID-19 spike protein attachment to hACE2 would potentially lead to better treatment against this disease.

Genus *Ulva* belongs to the family Ulvaceae which is comprised of cosmopolitan and abundant green macroalgae (approximately 100 species) that inhabit freshwater as well as saline shallow environments [7,8,9]. This algal genus is used as a source of traditional food in many Asian countries. In addition, several biological investigations revealed that *Ulva* can be of potential interest for the development of novel drugs and functional foods [9,10]. *Ulva* spp. were proved to possess diverse pharmacological effects that are ascribed to their chemical constituents which are mainly fatty acids, ceramides, terpenes, phenolics [10] and most importantly, sulphated polysaccharides known as ulvans. These sulfated polysaccharides were extracted from different *Ulva* species and displayed significant bioactivities in both in vitro and in vivo studies such as immune modulatory, anti-inflammatory, anticoagulant, antihyperlipidemic, cytotoxicity and antiviral potential against a broad range of enveloped and non-enveloped viruses [11]

*U. lactuca* is an edible green seaweed often known as sea lettuce. It grows on reef flats and rocky shores in the lower intertidal zone affected by waves [12,13,14]. Phytochemical investigations of *U. lactuca* resulted in the isolation of several bioactive phytoconstituents such as (+)-Epiloliolide, a carotenoid derivative which exhibited an apoptotic effect via the regulation of the p53 gene [13], an anti-inflammatory pyrone analogue of benzochromene named ulvapyrone [15] and 3-*O-β*-D glucopyranosyl-stigmasta-5,25-dien which has displayed antibacterial and antifungal effects [16]. In addition, palmitic acid, isofucosterol, hydrocarbons and norterpenes were also reported in *U. lactuca* [17,18]. It is noteworthy that the crude extracts of different *Ulva* species (including *U. lactuca*) exhibited significant antiviral activity against various DNA and RNA viruses [19,20,21,22,23,24,25,26,27,28,29]. Furthermore, the antiviral effect of ceramides and their derivatives was proven [30,31,32,33]. These findings and observations make *U. lactuca* a potential natural remedy for curing respiratory viral infections. Therefore, the purpose of this study was to isolate bioactive natural compounds from *U. lactuca* with promising antiviral activity against SARS-CoV-2 in addition to the rapid identification of its metabolites using liquid chromatography coupled to electrospray ionization high-resolution mass spectroscopy (LC-ESI-HRMS) technique. 

## 2. Results and Discussion

### 2.1. Metabolic Profiling

The metabolic profiling of the crude extract of *U. lactuca*, performed using LC-ESI-HRMS (Figures S1 and S2), showed the presence of a panel of diverse metabolites such as fatty acids, glycerides, sterols and oxysterols, ceramides, terpenoids, carbohydrates and amino acids (Table 1). These metabolites were detected by comparing their exact masses, with those recorded in databases, e.g., the Dictionary of Natural Products (DNP) and Metabolite and Chemical Entity (METLIN). Mass accuracy was determined by ((detected mass–expected mass)/expected mass) × 10^6^ and expressed in parts per million (ppm) error [34]. Previous assessment of the antiviral activity of various fatty acids revealed that medium-chain saturated and long-chain unsaturated fatty acids were highly active against enveloped viruses such as *Vesicular stomatitis* virus and *Herpes simplex* virus. Additionally, the monoglycerides of these fatty acids showed high antiviral activity at a concentration 10 times lower than that of free fatty acids. It was suggested that these antiviral fatty acids were able to disturb the viral envelope, causing leakage or a complete disintegration of the envelope and consequently the viral particles [35]. Moreover, some sterols have been shown to possess viral inhibition activity such as stigmasterol, which was effective against tobamoviruses with a virus inhibitory activity of 64% [36]. Ingallinella and coworkers [37] reported that the addition of a cholesterol group to enfuvirtide, Human Immune Virus -1 (HIV-1) peptide fusion inhibitor, potentiates its antiviral activity with IC_90_ values 300-fold lower than that of enfuvirtide alone. Oxysterols are cholesterol derivatives that contain an additional hydroxyl, epoxide or ketone group in the sterol nucleus and/or in the side chain, which have demonstrated antiviral activity by the inhibition of the viral replication of viruses with an external lipid membrane [38]. It is worth mentioning that oxysterols with a hydroxyl group at C-25 may inhibit the replication of hepatitis C virus, while oxysterol containing a hydroxyl group in its side chain, namely 24- hydroxycholesterol, may inhibit the murine cytomegalovirus and *Herpes simplex* virus type 1. With the additional oxygenation or hydroxylation of ring B, 7-ketone, 7*α*-OH and 7*β*-OH are also endowed with some antiviral activity [38]. Several reports have also stated that ceramide accumulation through a de novo biosynthesis pathway plays an antiviral and a protective role against influenza A/H1N1 virus infection [39]. The previously isolated ceramides were shown to exhibit antiviral activity against the pathogenic H5N1 avian influenza strain [40]. Based on these findings, the previously reported antiviral activity of different *Ulva* spp. including *U. lactuca* [19,20,21,22,23,24,25,26,27,28,29] may be attributed to the diverse chemical entities that were detected in this study using the LC-ESI-HRMS technique in addition to the polysaccharides previously extracted from *Ulva* spp. [11].

### 2.2. Isolation of Compounds **1**–**6**

**Figure 1:**
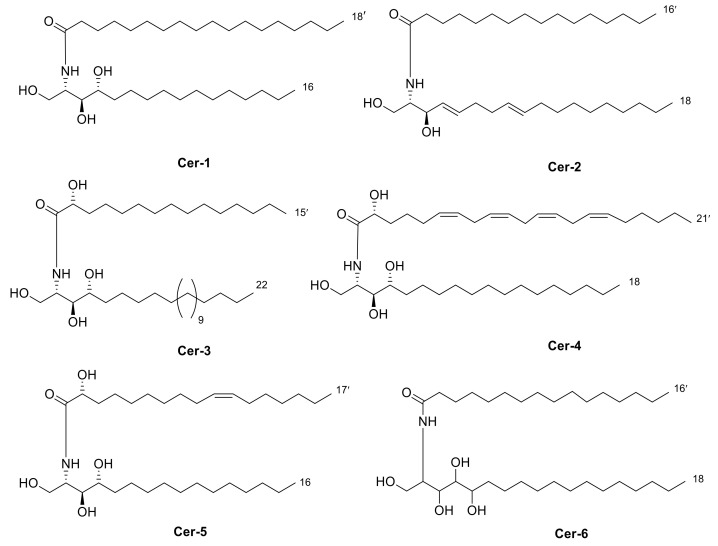
Chemical structures of isolated compounds **1–6**.

Four new ceramides in addition to two known ones (Figure 1) were isolated and purified from the fractions of the crude extract of *U. lactuca,* and the structures of the isolated compounds were elucidated based on spectroscopic data and by comparison with the data reported in the literature.

### 2.3. Identification of Isolated Compounds

Compound **1** (Figure 1) was isolated as a white powder. It has a molecular formula of C_34_H_70_NO_4_ derived from its positive HRESIMS pseudo molecular ion at *m/z* 556.5660 [M + H]^+^ (Figure S3) (calculated for 556.5305), representing one degree of unsaturation. The ^1^H spectrum (C_5_D_5_N, 400 Hz) of compound **1** is listed in Table 2 using deuterated pyridine as a solvent (Figure S4). The spectrum manifested resonances of an amide proton at *δ*_H_ 8.50 (d, *J* = 8 Hz) as well as overlapped peaks of protons of a long methylene chain at *δ*_H_ 1.21–1.26, representing a ceramide skeleton. Moreover, resonances of the long-chain base of 2-amino-1,3,4-triol were observed at *δ*_H_ 4.95 (m), 5.07 (m), 4.38 (m), and 4.27 (m) assigned to H-1, H-2, H-3 and H-4, respectively. Resonances of terminal methyl groups at *δ*_H_ 0.84 (t, *J* = 8 Hz) were assigned to CH_3_-16 and CH_3_-18′. On the other hand, the ^13^C NMR spectrum (C_5_D_5_N, 100 Hz) (Table 3) (Figures S5 and S6) showed characteristic resonances of a 2-amino-1,3,4-triol unit of the long-chain base at *δ*_C_ 62.2 (C-1), 53.8 (C-2), 76.7 (C-3) and 73.1 (C-4), while an amide carbonyl group was assigned at *δ*_C_ 173.5 (C-1′). In addition, the terminal methyl groups were detected at *δ*_C_ 14.2 (C16 and C18′) and carbons of long methylene chain were detected at *δ*_C_ 29.6–29.9. GC–MS analysis of the fatty acid methyl esters of compound **1** was performed after its methanolysis showing a peak with a molecular ion of *m/z* 298 which corresponded to the methyl ester of octadecanoic acid, C _(18:0)_. (Figure S7). The configuration of the ceramide moieties was determined by f ^13^C NMR comparison with analogs as reported in the literature [49]. Thus, the structure of **1** was determined to be N-[(2*S*,3*R*,4*R*)-1,3,4-trihydroxy-hexadecan-2-yl] octadecanamide. To the best of our knowledge, this compound is considered a new compound. Henceforth, it is denoted as **Cer-1**.

Compound **2** (Figure 1) was identified as N-[1,3-dihydroxy-octadeca-4,8-diene] hexadecanamide by comparing its spectral data (Table 2 and Table 3) with those reported in the literature [33]. It is worth mentioning that this ceramide was previously isolated from *Ulva fasciata* and showed antiviral activity against Japanese encephalitis virus [33]. Henceforth, it is denoted as **Cer-2**.

Compound **3** (Figure 1) was obtained as a white powder, and its molecular formula was determined to be C_37_H_76_NO_5_, as obtained from its HRESIMS pseudo molecular ion at *m/z* 614.5067 [M + H]^+^ (Figure S8) (calculated for 614.5723), representing one degree of unsaturation. The ^1^H NMR (C_5_D_5_N, 400 Hz) and ^13^C NMR (C_5_D_5_N, 100 Hz) spectral data of compound **3** are listed in Table 2 and Table 3, respectively, using deuterated pyridine as a solvent (Figures S9 and S10). The spectrum also manifested the sphingolipid skeleton as mentioned above. In addition, the resonance of alpha hydroxy fatty acid was determined at *δ*_H_ 4.60 (t, *J* = 8 Hz) and *δ*_C_ 72.1 (C-2′). Compound **3** was subjected to hydrolysis then oxidation, using aqueous HCl/MeOH for methanolysis giving an α-hydroxy fatty acid methyl ester and a phytosphingosine base. Then, the α-hydroxy fatty acid methyl ester was converted into a fatty acid methyl ester via oxidative cleavage using KMnO_4_ and NaIO_4_ [40]. The resultant fatty acid methyl ester was analyzed by GC–MS which demonstrated a molecular ion peak of *m/z* 242 corresponding to a C14 fatty acid methyl ester (Figure S11). The assignment of the configuration of the ceramide moiety was accomplished through x ^13^C NMR data comparison with analogs as reported in the literature [50]. Thus, the structure of **3** was determined to be 2-hydroxyN-[(2*S*,3*R*,4*R*)-1,3,4-trihydroxy-docosan-2-yl] pentadecanamide. To the best of our knowledge, this compound is also considered a new compound. Henceforth, it is denoted as **Cer-3**.

Compound **4** (Figure 1) was obtained as a white powder, and its molecular formula was determined to be C_39_H_71_NNaO_5_, as established from its HRESIMS pseudo molecular ion at *m/z* 656.5080 [M + Na]^+^ (Figure S12) (calculated for 656.5230), representing five degrees of unsaturation. The ^1^H spectrum (CDCl_3_, 400 Hz) of compound **4** is listed in Table 2 using deuterated chloroform as a solvent (Figure S13). Additionally, the resonance of *α* hydroxy fatty acid was determined at *δ*_H_ 4.23 (t, *J* = 8 Hz). Resonances of the long-chain base of 2-amino-1,3,4-triol were observed as mentioned before, while multiplied olefinic protons were determined at *δ*_H_ 5.32–5.81. On the other hand, the ^13^C NMR spectrum (CDCl_3_, 100 Hz) (Table 3) (Figures S14 and S15) showed typical signals of 2-amino-1,3,4-triol in addition to olefinic carbons at *δ*_C_ 126, 129, 132 and 134. Compound **4** was subjected to methanolysis followed by oxidation resulting in a fatty acid methyl ester which was inspected by GC/MS, showing a molecular ion peak of *m/z* 318 which corresponded to the methyl ester of arachidonic acid, C_(20:4)_ (Figure S16). Thus, the structure of **4** was set to be 2-hydroxy-N-[(2*S*,3*R*,4*R*)-1,3,4-trihydroxy-octadecan-2-yl] henicosa-6,9,12,15-tetraene-amide [51]. To the best of our knowledge, this compound is considered a new compound. Henceforth, it is denoted as **Cer-4**.

Compound **5** (Figure 1) was obtained as a white powder, and its molecular formula was determined to be C_33_H_65_NNaO_5_, as derived from its HRESIMS pseudo molecular ion at *m/z* 578.4604 [M + Na]^+^ (Figure S17) (calculated for 578.4760), representing two degrees of unsaturation. The ^1^H NMR (C_5_D_5_N, 400 Hz) and ^13^C NMR spectral data (C_5_D_5_N, 100 Hz) of compound **3** are listed in Table 2 and Table 3, respectively, showing a ceramide nucleus (Figures S18 and S19). Herein, the olefinic carbons were detected at *δ*_C_ 132.7. It is well established that double bond geometry in a long-chain alkene can be deduced from the ^13^C-NMR chemical shift of the allylic carbon. In the (*Z*) isomer, the allylic carbon resonance was observed close to *δ*_C_ 27, while in the (*E*) isomer, its chemical shift is approximately *δ*_C_32 [52]. Therefore, the olefinic group is determined to be *Z*-type. The GC–MS analysis of the fatty acid methyl esters resulted from compound **5** methanolysis and oxidation which displayed a molecular ion peak of *m/z* 268 corresponding to 9*Z*-hexadecenoic acid, C _(16:1)_ methyl ester (Figure S20). The configuration of the ceramide part of the compound was determined by ^13^C NMR comparison with analogues mentioned in the literature [53]. Thus, the structure of **5** was determined to be 2- hydroxy-N-[(2*S*,3*R*,4*R*)-1,3,4-trihydroxy-hexadecan-2-yl]-10-heptadecenamide. To the best of our knowledge, this compound is also considered a new compound. Henceforth, it is denoted by **Cer-5**.

Compound **6** (Figure 1) was identified as N-[1,3,4,5-tetrahydroxy-octadecan-2-yl] hexadecanamide by comparing its spectral data (Table 2 and Table 3) with those reported in the literature. It is worth noting that this antiviral ceramide was also previously isolated from *Ulva fasciata* [54]. Henceforth, it is denoted as **Cer-6**.

### 2.4. Docking Studies

#### 2.4.1. Molecular Docking Simulation of Isolated Ceramides and Detected Metabolites by LC-ESI-HRMS Technique to Site 4 of hACE2–SARS-CoV-2 Spike Protein Complex

The outbreak of SARS-CoV-2 is currently a global disaster [55], as confirmed cases exceed 250 million in addition to more than 5 million deaths all over the world [56]. Unfortunately, to date, there is no validated treatment for this infection despite several ongoing clinical and preclinical studies [55]. The experimental procedure to obtain a de novo drug is expensive (costing millions of dollars) and long (requiring decades)—and is thus not a solution in the present emergency situation. Thus, in silico studies are playing a significant role in accelerating research to discover potential leads against SARS-CoV-2. Drug repurposing including those of natural origin together with molecular docking and dynamic simulation are the main strategies currently applied for the development of antiviral therapies for the treatment of COVID-19 [57,58,59]. Moreover, molecular dynamic simulation investigations help validate the binding between the protein and the ligand. Such techniques can be employed in drug developmental studies that further help in drug optimization with improved selectivity and specificity [57]. 

At the present time, numerous natural products are being evaluated for possible antiviral potential against SARS-CoV-2 [55,56,57]. In addition, evidence of the antiviral activities of algae belonging to genus *Ulva* against both RNA and DNA viruses have been acquired [19,20,21,22,23,24,25,26,27,28,29]. 

Based on the aforementioned considerations, the phytoconstituents of *Ulva lactuca* were evaluated by in silico studies as possible leads against SARS-CoV-2

Protein–protein docking demonstrated that SARS-CoV-2 spike proteins possess a strong affinity for hACE2 [6]. Although, many compounds have been shown using virtual screening to inhibit ACE2, these compounds may not be useful for treating SARS-CoV-2 infection because hACE2 is considered a protective factor against lung injury [60]. Only compounds that are able to prevent protein–protein interaction between the spike protein and ACE are considered potential therapeutic agents. One such compound that could target the binding interface between spike protein and ACE2 is hesperidin. It was suggested that the interaction of ACE2 with RBD might be disrupted by hesperidin. Since a clear interlock of the ACE2 interface with hesperidin was noticed by superimposing the ACE2–receptor binding domain (RBD) complex on the hesperidin—RBD complex [61,62]. Hesperidin was predicted to be on the middle shallow pit of the receptor-binding domain (RBD) surface of the spike protein [61]. Thus, this compound in particular was utilized as a reference in the present study.

**Figure 2:**
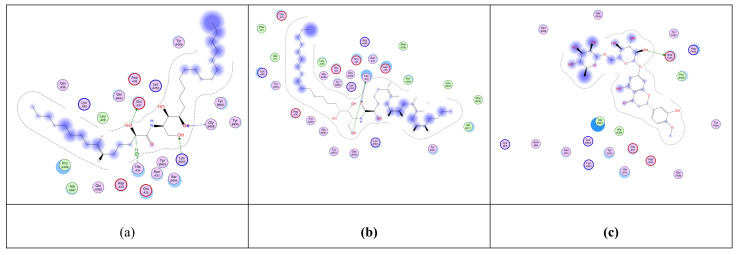
2D representation of the docking of compounds **Cer-5** (**a**); **Cer-4** (**b**); and hesperidin (**c**) into the SARS-CoV-2 spike binding site with hACE2.

**Figure 3:**
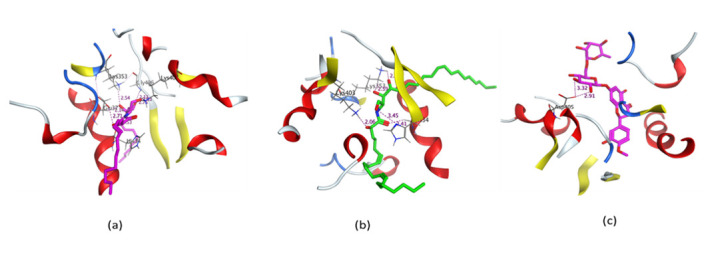
3D representation of the docking of compounds **Cer-5** (**a**); **Cer-4** (**b**); and hesperidin (**c**) into the SARS-CoV-2 spike binding site with hACE2.

The crystal structure of the RBD of the spike protein of SARS-CoV-2 in the complex with hACE2 was determined by Shang and collaborators. An hACE2-binding ridge in SARS-CoV-2 RBD possesses a tighter conformation compared to the SARS-CoV-2 RBD. Furthermore, two virus-binding hotspots at the RBD–hACE2 interface were stabilized by numerous residues’ changes in the SARS-CoV-2 RBD. Consequently, the binding affinity of SARS-CoV-2 RBD with hACE2 increased. [63]. We used the crystallographic model for docking studies with the isolated ceramides (**Cer-1**–**6**) and the metabolites. Molecular docking simulation results as well as the interaction energies of the ceramides, the metabolites (compounds **1**–**17**) and the reference hesperidin with SARS-CoV-2 spike protein- hACE2 complex are shown in Table 4. All the ceramide compounds and compounds **8** and **16** showed excellent binding energies with hACE2–spike protein compared to hesperidin, while compounds **2**–**4**, **10**,**11**,**13**,**17** showed weaker binding energies than hesperidin. The ceramide compounds **Cer-4** and **Cer-5** were the most promising compounds and their binding mode with the hACE2–spike protein complex is shown in Figure 2 and Figure 3.

#### 2.4.2. Molecular Docking Simulation of Isolated Ceramides and Detected Metabolites by LC-ESI-HRMS Technique to SARS-CoV-2 M^pro^

**Figure 4:**
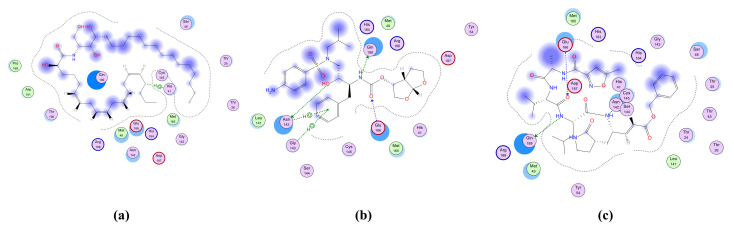
2D representation of the docking of compounds **Cer-4** (**a**); darunavir (**b**); and **N3** (**c**) into the SARS-CoV-2 M^pro^.

**Figure 5:**
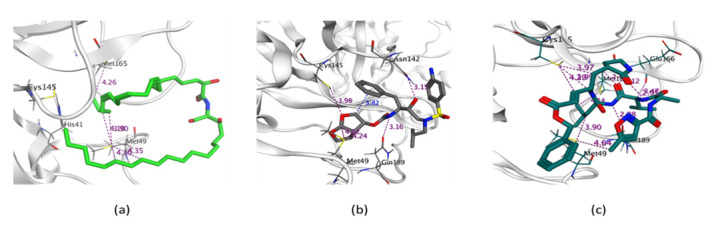
3D representation of the docking of compounds **Cer-4** (**a**); darunavir (**b**); and **N3** (**c**) into the SARS-CoV-2 M^pro^.

The proteinase (M^pro^)is a key enzyme which plays an essential role in SARS-CoV-2 replication and transcription and its closely related homologues in humans are absent. Therefore, M^pro^ becomes a druggable e-target for the development of antivirals against SARS-CoV-2 [64]. Drugs that inhibit M^pro^ are expected to play a crucial role in restricting viral transcription and replication [65,66]. The (M^pro^) is specifically involved in proteolytic cleavages of polyproteins to release functional polypeptides. This enzyme digests the polyprotein of at least 11 conserved sites, commencing with the autolytic cleavage from pp1a and pp1ab of the enzyme itself [67]. The M^pro^ owns a Cys–His catalytic dyad, and the substrate-binding site is present in a cleft between Domains I and II. The crystal structure of M^pro^ in the complex with a known inhibitor, N3, was determined by Zhenming and coworkers [68], where N3 is inside the substrate-binding pocket. The molecular simulation of the six isolated ceramides, compounds **1**–**17**, and the positive controls N3 and darunavir in the active site of M^pro^ was performed. All the compounds showed nearly identical bindings such as N3 with interaction energies shown in Table 5. The compounds were stabilized at the N3-binding site of M^pro^ by many different types of electrostatic interactions. All the ceramide compounds (**Cer-1**–**6**) showed excellent binding energies with the best being **Cer-4** (94% that of N3) (Figure 4 and Figure 5). Ceramides (**Cer-1**–**6**) and compounds **12**, **8**, **9**, **15**, **16** also demonstrated higher binding energies compared to darunavir. The other compounds showed only moderate binding energies. 

#### 2.4.3. ADME Studies

The evaluation of the in silico drug-likeness and pharmacokinetics of biomolecules is a rapid predictive and cost-effective technique in drug discovery. The studied molecules must demonstrate significant biological activity and low toxicity as well. In addition, the accessibility and concentration of the biologically active molecule at the therapeutic target in the organism is equally essential. It has been demonstrated that the determination of the ADME properties of a potential drug candidate in the discovery phase drastically decreases the chances of drug failure in the clinical phases due to its pharmacokinetic characters [69]. ADME computational models have been recognized as an acceptable alternative to experimental procedures for the prediction of drug pharmacokinetics, particularly at first steps, when studied chemical entities are immense but the availability of such compounds is rare [70]. The topological polar surface area (TPSA), considering sulfur and phosphorus as polar atoms [71], has been a valid descriptor used in the fast estimation of some ADME properties, concerning the crossing of biological barriers such as absorption and brain access [72]. Studying the physicochemical properties and ADME of the six isolated ceramides (Cer-**1**–**6**), compounds **1**–**17**, hesperidin, darunavir and N3 (Table 6) revealed that all compounds have a reasonable TPSA, i.e., between 20 and 130 Å2 except for compounds **8**, **9** and the references’ compounds. The most promising compounds are predicted to have low absorption from GIT. However, this can be overcome by converting them into prodrugs through the esterification of their hydroxyl groups. An MLOGP of less than 4.15 suggests reasonable lipophilicity suggestive of oral bioavailability. Compounds **Cer-5**, **Cer-6**, **1**, **2**, **7**–**9**, **12**, **15**–**17** and the reference compounds were of reasonable lipophilicity. The predicted BBB permeability is shown in Table 6, indicating the most promising compounds (**Cer-1–6**) and all compounds except compounds **1**–**3** will not cause CNS side effects. Noteworthily, all test compounds showed no PAINS (pan assay interference compounds) alert, which reveals that all compounds do not contain pharmacologically unwanted moieties in their chemical structures that could be liberated into the metabolism.

## 3. Materials and Methods 

### 3.1. Plant Material

The green algae *U. lactuca* was collected from Safaga on the coast of the Red Sea and was kept in sea water containing plastic bags to prevent evaporation. Then, epiphytes and rock debris were separated. Samples were rinsed with fresh water for the removal of surface salts. Then, samples were air-dried and stored at a low temperature (−24 °C) until further processing. The alga was identified by morphological techniques according to [73,74] by Dr. Tarek Temraz, Marine Science Department, Faculty of Science, Suez Canal University, Ismailia, Egypt. A voucher specimen under registration no. SAA-131 was placed in the Pharmacognosy Department herbarium, Faculty of Pharmacy, Suez Canal University, Ismailia, Egypt.

### 3.2. General Experimental Procedure

A Bruker Avance III HD 400 spectrometer operating at 400 MHz was utilized to obtain ^1^H and ^13^C NMR spectra. The chemical shifts of ^1^H and ^13^C NMR are expressed in *δ* (ppm) values regarding the solvent peaks *δ*_H_ 7.26 and *δ*_C_ 77 ppm for CDCl_3_, and also *δ*_H_ 7.19, 7.55, 8.71 and *δ*_C_ 123.5, 135.5, 149.2 ppm for C_5_D_5_N. The coupling constants are obtained in Hertz (Hz). Aluminum-backed plates pre-coated with silica gel F254 (20 × 20 cm; 200 µm; 60 Å (Merck^™^, Darmstadt, Germany) were used for TLC analysis. For column chromatography, silica gel 60/230–400 µm mesh size (Whatman^™^, Sanford, ME, USA) was utilized. C18- reversed phase octadecyl silica (ODS) gel (Fluka™, Buchs, Switzerland) and Sephadex^®^ LH-20 (Sigma Aldrich^®^, Bremen, Germany) were also used for final purification.

### 3.3. Metabolic Profiling 

The metabolic study was carried out using LC-ESI-HRMS for the rapid identification of different primary and secondary metabolites, as previously described in detail [34]. The identification of the phytochemicals was achieved by the comparison of their spectral data, accurate masses in particular, with those from some databases such as METLIN and DNP.

### 3.4. Extraction and Isolation

The air-dried material of *U. lactuca* (90 g) was milled and then extracted at room temperature with methanol (MeOH, 2 L × 3). The obtained extracts were combined together and then concentrated in vacuo to yield a dark-green residue (Ulv, 23 g) which was dissolved in a MeOH:H_2_O (6:4) solvent system and then fractionated by partitioning with different solvents in increasing order of polarity: *n*-hexane, chloroform, ethyl acetate (EtOAc) and *n*-butanol. The EtOAc fraction (Ulv-EA, 5.79 g) was chromatographed on a silica gel column using *n*-hexane: EtOAc: MeOH (80:20:0) up to (0:75:25) and step-by-step gradient elution, giving five subfractions: Ulv-EA-1–Ulv-EA-5. Subfraction Ulv-EA-3 (330 mg) was rechromatographed on silica gel using hexane: EtOAc: methanol (50:50:0) up to (0:90:10) giving five subfractions: Ulv-EA-3-1′–Ulv-EA-3-5′. One of the resulting subfractions (Ulv-EA-3-2′, 43 mg) was purified using octadecyl silica gel column chromatography (ODS column) and methanol: isopropanol (60:40) as an eluent giving compound **1** (13 mg) and compound **2** (11 mg). Another subfraction (Ulv-EA-3-3′, 169 mg) was rechromatographed on silica gel column using EtOAc: methanol (100:0 to 90:10), giving three subfractions: Ulv-EA-3-3′-1–Ulv-EA-3-3′-3. Among them, subfraction Ulv-EA-3-3′-2 (67 mg) was rechromatographed on a sephadex LH-20 column eluted with CHCl_3_-MeOH (1:1) giving 2 subfractions: Ulv-EA-3-3′-2-1 and Ulv-EA-3-3′-2-2. The first one (EA-3-3′-2-1, 23 mg) was applied to an ODS column using methanol: isopropanol (70:30) to afford **3** (6 mg), **4** (7 mg), while the second one (EA-3-3′-2-2, 27 mg) was also applied to an ODS column using methanol: isopropanol (70:30) to afford **5** (6 mg) and **6** (9 mg).

### 3.5. Docking Studies

#### 3.5.1. Test Compounds Preparation

After constructing the test compounds as a 3D model and formal charges on atoms were implemented by a 2D model, a conformational search was performed to the test compounds. Energy minimizations were applied to the RMSD gradient of 0.01 Kcal/mole and an RMS distance of 0.1 Å with an MMFF94X force-field with the automatic calculation of the partial charges. The chemical structures with energy minimization were saved as molecular data-based MDB files for docking studies.

#### 3.5.2. Optimization of the Enzymes’ Active Site

From the Protein Data Bank (PDB), the X-ray crystallographic structure of SARS-CoV-2 spike protein–hACE2 complex was downloaded through (http://www.rcsb.org/, PDB code 6VW1) (accessed on 30 August 2020). Additionally, M^pro^ complexed with N3 was downloaded (http://www.rcsb.org/, PDB code 6LU7) (accessed on 30 August 2020). The optimization of the proteins was achieved by adding hydrogen atoms and the automatic correction of atoms’ connections in the protein. Additionally, the atoms’ potentials were performed. For the identification of the active site of hACE2- SARS-CoV-2–spike protein complex, we utilized site finder. The largest site of the hACE2 complex with the SARS-CoV-2 spike protein was selected from the site finder of the pocket. However, for M^PRO^, the N3 binding site was selected for docking studies. 

#### 3.5.3. Docking of the Test Molecules to the Biggest Site of hACE2 Complex with SARS-CoV-2 Spike Protein and to SARS-CoV-2 M^pro^

The conformation of the database of the docking of the target compounds was performed. First, the active site file was loaded and then the docking tool was started. The adjustment of the program specifications to the biggest site of the hACE2 complex with the SARS-CoV-2 spike protein as the docking site (dummy atoms) was performed in addition to applying the alpha triangle as the placement methodology. The scoring methodology London d G was applied for default values. The target compounds’ MDB file was used and fitting calculations were automatically performed. The poses which demonstrated the best reciprocity between the ligand and the contact surface of the hACE2–SARS-CoV-2 spike protein complex were chosen and saved to calculate the binding energy.

#### 3.5.4. ADME Studies

The ADME of all the mentioned compounds of the test compounds isolated from *U. lactuca* and the reference compounds, hesperidin, darunavir and N3 were calculated by the charge-free website http://www.swissadme.ch (accessed on 5 September 2020).

## 4. Conclusions

We identified four new ceramides from the methanolic extract of the green algae *U. lactuca* in addition to two previously reported ceramides. Furthermore, various metabolites such as fatty acids, their glyceride derivatives, terpenoids, sterols and oxysterols were identified using the LC-ESI-HRMS technique. Molecular docking studies with the six isolated ceramides as well as all identified metabolites suggest that these compounds may inhibit viral attachment to host cells or viral replication and transcription. **Cer-4**, 2- hydroxy-N-[(2*S*,3*R*,4*R*)-1,3,4-trihydroxy-octadecan-2-yl] henicosa-6,9,12,15-tetraene-amide, was the most active compound of the hACE2–SARS-CoV-2 spike protein binding complex inhibition. It also showed the best binding affinity to the M^PRO^ N3 binding site. This study identified potential natural products that could be developed to treat the SARS-CoV-2 virus disease. Future extensive wet-lab in vitro and in vivo studies will be conducted to verify our findings.

## Figures and Tables

**Table 1:** Metabolic profiling (LC-ESI-HRMS) of methanolic crude extract of *U. lactuca*.

	Polarity Mode	Ret. Time (min)	*Precursor m/z*	MZmine ID	Detected Mass	Expected Mass	Mass Error (ppm)	Name	Structure	Reported Previously in	Ref.
1. Fatty acids											
**1**	Positive	7.14	249.1854	5976	248.1781	248.1776	2.01	Hexadeca-4,7,10,13-tetraenoic acid	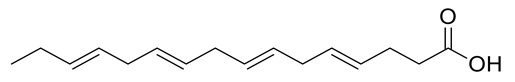	*Ulva fasciata*	[41]
**2**	Negative	10.13	295.2267	667	296.2340	296.2351	−3.71	11*E*-Oxo-octadec-12-enoic acid	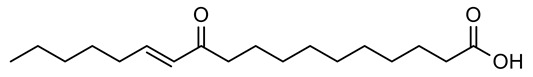	*Ulva fasciata*	[41]
**3**	Negative	10.52	275.2005	3655	276.2078	276.2089	−3.98	6*Z*,9*Z*,12*Z*,15*Z*-Octadecatetraenoic acid	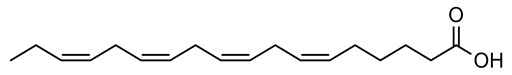	*Ulva fasciata*	[41]
**4**	Positive	11.04	305.2479	565	304.2406	304.2402	1.31	Arachidonic acid	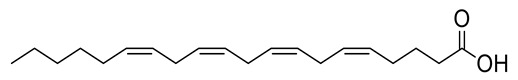	*Ulva lactuca*	[42]
**5**	Negative	11.15	277.2161	2459	278.2234	278.2246	−4.31	Linolenic acid	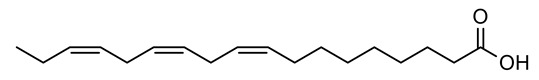	*Ulva fasciata*	[41]
**6**	Negative	11.84	283.2627	2827	284.2700	284.2715	−5.28	Stearic acid	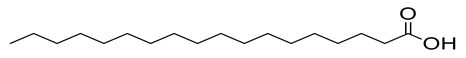	*Ulva fasciata*	[43]
2. Glycerol derivatives/glycerides											
**7**	Positive	8.97	331.2874	145	330.2774	330.2770	1.21	Glycerol monopalmitate	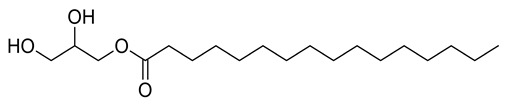	*Ulva prolifera*	[44]
**8**	Positive	9.40	521.3723	8312	520.3650	520.3611	7.49	1-*O*-Octadecanoic acid-3-*O-β*-D-galactopyranosyl glycerol	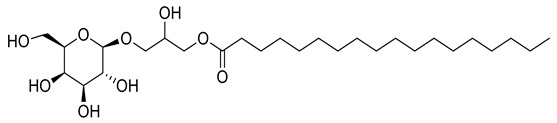	*Ulva prolifera*	[44]
**9**	Negative	10.02	491.3222	2431	492.3295	492.3298	−0.61	1-*O*-Palmitoyl-3-*O-β*-D-galactopyranosyl glycerol	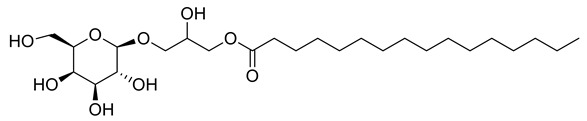	*Ulva prolifera*	[44]
3. Sterols and oxysterols											
**10**	Positive	10.41	443.3524	5136	442.3452	442.3447	1.13	5,28-Stigmastadiene-3*β*,24-diol-7-one	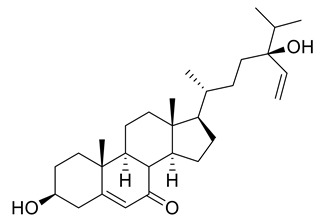	*Ulva australis*	[45]
**11**	Negative	13.85	395.3160	2931	396.3353	396.3392	−9.84	Ergosterol	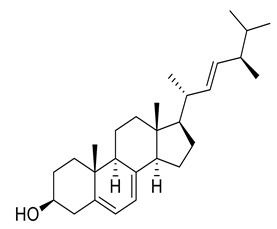	*Ulva pertusa*	[46]
**12**	Positive	15.25	575.4362	6572	574.4239	574.4233	1.04	3-*O-β*-D Glucopyranosyl-clerosterol	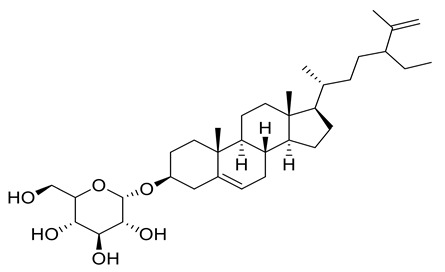	*Ulva lactuca*	[16]
**13**	Positive	16.52	429.3715	5560	428.3643	428.3654	−2.57	24,28-Epoxy-24-ethylcholesterol	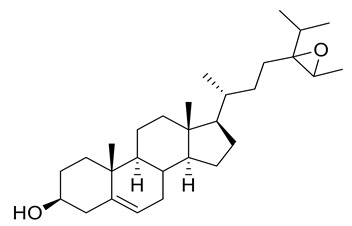	*Ulva australis*	[45]
**14**	Positive	16.52	429.3715	5560	428.3643	428.3654	−2.57	5,28-Stigmastadiene-3*β*,24-diol	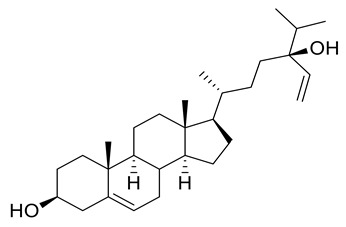	*Ulva australis*	[45]
4. Ceramide and sphingoid base											
**15**	Positive	12.34	328.3198	6410	327.3125	327.3137	−3.67	N,N-Dimethyl sphingosine	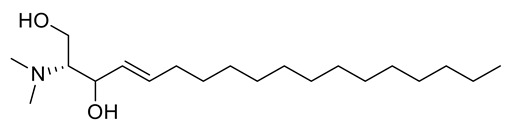	fungi	[42]
**16**	Positive	14.35	558.5128	7471	557.5055	557.5019	6.46	N-[pentadecanoate ]-1,3,4,5-tetrahydroxy-2-amino-octadecane	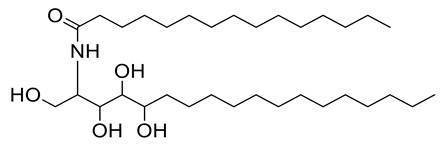	*Ulva fasciata*	[47]
5. Terpenoid											
**17**	Positive	9.06	223.2061	5846	222.1988	222.1984	1.80	2,5,5-Trimethyl-4-(4′-methyl-3′-pentenyl)-2-cyclohexen-1-ol	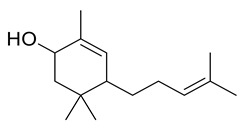	*Ulva fasciata*	[48]

**Table 2:** ^1^H NMR spectra of isolated compounds **1–6**.

	Cer-1 *	Cer-2 *	Cer-3 *	Cer-4 ^#^	Cer-5 *	Cer-6 *
1	4.95 (m)	4.53 (m)	4.42 (m)	4.06 (m)	4.60 (m)	4.54 (m)
2	5.07 (m)	5.04 (m)	5.06 (m)	5.08 (m)	5.12 (m)	5.11 (m)
3	4.38 (m)	4.94 (m)	4.39 (m)	3.87 (m)	4.27 (m)	4.44 (m)
4	4.27 (m)	5.46 (m)	4.31 (m)	3.75 (m)	4.44 (m)	4.41 (m)
5	2.22 (m)	5.46 (m)	2.18 (m)	1.99 (m)	2.10 (m)	4.43 (m)
6	1.97 (m)	2.19 (m)	1.10–1.26 (m)	1.28–1.36 (m)	1.22–1.28 (m)	2.02 (m)
7	1.21–1.26 (m)	1.94 (m)	1.10–1.26 (m)	1.28–1.36 (m)	1.22–1.28 (m)	1.25–1.30 (m)
8	1.21–1.26 (m)	5.46 (m)	1.10–1.26 (m)	1.28–1.36 (m)	1.22–1.28 (m)	1.25–1.30 (m)
9	1.21–1.26 (m)	5.46 (m)	1.10–1.26 (m)	1.28–1.36 (m)	1.22–1.28 (m)	1.25–1.30 (m)
10	1.21–1.26 (m)	1.90 (m)	1.10–1.26 (m)	1.28–1.36 (m)	1.22–1.28 (m)	1.25–1.30 (m)
11	1.21–1.26 (m)	1.23–1.29 (m)	1.10–1.26 (m)	1.28–1.36 (m)	1.22–1.28 (m)	1.25–1.30 (m)
12	1.21–1.26 (m)	1.23–1.29 (m)	1.10–1.26 (m)	1.28–1.36 (m)	1.22–1.28 (m)	1.25–1.30 (m)
13	1.21–1.26 (m)	1.23–1.29 (m)	1.10–1.26 (m)	1.28–1.36 (m)	1.22–1.28 (m)	1.25–1.30 (m)
14	1.67 (m)	1.23–1.29 (m)	1.10–1.26 (m)	1.28–1.36 (m)	1.22–1.28 (m)	1.25–1.30 (m)
15	1.35 (m)	1.23–1.29 (m)	1.10–1.26 (m)	1.28–1.36 (m)	1.36 (m)	1.25–1.30 (m)
16	0.84 (t, *J* = 8)	1.23–1.29 (m)	1.10–1.26 (m)	1.28–1.36 (m)	0.85 (t, *J* = 8)	1.25–1.30 (m)
17	-	1.68 (m)	1.10–1.26 (m)	1.28–1.36 (m)	-	1.71 (m)
18	-	0.84 (t, *J* = 8)	1.10–1.26 (m)	0.84 (t*, J* = 8)	-	0.85 (t, *J* = 8)
19	-	-	1.10–1.26 (m)	-	-	-
20	-	-	1.10–1.26 (m)	-	-	-
21	-	-	1.70 (m)	-	-	-
22	-	-	0.81 (t, *J* = 4)	-	-	-
1′	--	-	-	-	-	-
2′	2.45 (t, *J* = 8)	4.19 (brt, *J* = 4)	4.60 (t, *J* = 8)	4.23 (t, *J* = 8)	4.97 (t, *J* = 8)	2.23 (brt, *J* = 4)
3′	1.84 (m)	1.98 (m)	1.95 (m)	1.95 (m)	2.10 (m)	1.71 (m)
4′	1.21–1.26 (m)	1.23–1.29 (m)	1.10–1.26 (m)	1.28–1.36 (m)	1.22–1.28 (m)	1.25–1.30 (m)
5′	1.21–1.26 (m)	1.23–1.29 (m)	1.10–1.26 (m)	1.28–1.36 (m)	1.22–1.28 (m)	1.25–1.30 (m)
6′	1.21–1.26 (m)	1.23–1.29 (m)	1.10–1.26 (m)	5.77 (m)	1.22–1.28 (m)	1.25–1.30 (m)
7′	1.21–1.26 (m)	1.23–1.29 (m)	1.10–1.26 (m)	5.77 (m)	1.22–1.28 (m)	1.25–1.30 (m)
8′	1.21–1.26 (m)	1.23–1.29 (m)	1.10–1.26 (m)	1.28–1.36 (m)	1.22–1.28 (m)	1.25–1.30 (m)
9′	1.21–1.26 (m)	1.23–1.29 (m)	1.10–1.26 (m)	5.53 (m)	2.26 (m)	1.25–1.30 (m)
10′	1.21–1.26 (m)	1.23–1.29 (m)	1.10–1.26 (m)	5.53 (m)	5.5 (m)	1.25–1.30 (m)
11′	1.21–1.26 (m)	1.23–1.29 (m)	1.10–1.26 (m)	1.28–1.36 (m)	5.5 (m)	1.25–1.30 (m)
12′	1.21–1.26 (m)	1.23–1.29 (m)	1.10–1.26 (m)	5.45 (m)	2.26 (m)	1.25–1.30 (m)
13′	1.21–1.26 (m)	1.23–1.29 (m)	1.10–1.26 (m)	5.45 (m)	1.22–1.28 (m)	1.25–1.30 (m)
14′	1.21–1.26 (m)	1.23–1.29 (m)	1.70 (m)	1.28–1.36 (m)	1.22–1.28 (m)	1.25–1.30 (m)
15′	1.21–1.26 (m)	1.68 (m)	0.81 (t, *J* = 4)	5.34 (m)	1.22–1.28 (m)	1.71 (m)
16′	1.67 (m)	0.84 (t, *J* = 8)	-	5.34 (m)	1.36 (m)	0.85 (t, *J* = 8)
17′	1.35 (m)	-	-	1.28–1.36 (m)	0.85 (t, *J* = 8)	-
18′	0.84 (t, *J* = 8)	-	--	1.28–1.36 (m)	-	-
19′	-	-	-	1.28–1.36 (m)	-	-
20′	-	-	-	1.74 (m)	-	-
21′	-	-	-	0.84 (t, *J* = 8)	-	-
NH	8.50 (d, *J* = 8)	8.57 (d, *J* = 12)	8.52 (d, *J* = 12)	7.25 (d, *J* = 12)	8.58 (d, *J* = 8)	8.55 (d, *J* = 12)

*: ^1^H NMR spectra using deuterated pyridine (C_5_D_5_N) as a solvent; #: ^1^H NMR spectra using deuterated chloroform (CDCl_3_) as a solvent.

**Table 3:** ^13^C NMR spectra of isolated compounds **1–6**.

	Cer-1 *	Cer-2 *	Cer-3 *	Cer-4 ^#^	Cer-5 *	Cer-6 *
1	62.2	62.6	61.7	61.9	61.7	61.9
2	53.8	54.5	52.6	54.4	52.6	52.9
3	76.7	75.1 (72)	76.5	77.2	76.4	72.4
4	73.1	131.1	72.7	74.0	72.7	73.0
5	33.9	132.0	33.8	32.5	33.8	76.6
6	29.6–29.9	32.9	29.2–30.0	27.3	29.3–29.6	34.0
7	29.6–29.9	28.0	29.2–30.0	29.3–29.7	29.3–29.6	29.5–30.2
8	29.6–29.9	131.9	29.2–30.0	29.3–29.7	29.3–29.6	29.5–30.2
9	29.6–29.9	129.9	29.2–30.0	29.3–29.7	29.3–29.6	29.5–30.2
10	29.6–29.9	39.9	29.2–30.0	29.3–29.7	29.3–29.6	29.5–30.2
11	29.6–29.9	29.5–29.9	29.2–30.0	29.3–29.7	29.3–29.6	29.5–30.2
12	29.6–29.9	29.5–29.9	29.2–30.0	29.3–29.7	29.3–29.6	29.5–30.2
13	29.6–29.9	29.5–29.9	29.2–30.0	29.3–29.7	29.3–29.6	29.5–30.2
14	29.6–29.9	29.5–29.9	29.2–30.0	29.3–29.7	29.3–29.6	29.5–30.2
15	22.9	29.5–29.9	29.2–30.0	29.3–29.7	31.8	29.5–30.2
16	14.2	31.9	29.2–30.0	31.6	13.9	32.0
17	-	22.8	29.2–30.0	22.6	-	22.6
18	-	14.1	29.2–30.0	14.0	-	14.1
19	-	-	29.2–30.0	-	-	-
20	-	-	29.2–30.0	-	-	-
21	-	-	31.8	-	-	-
22	-	-	13.9	-	-	-
1′	173.5	175.7	175.1	175.7	175.1	175.3
2′	36.9	35.6	72.1	72.5	72.1	35.6
3′	29.6–29.9	25.7	35.3	34.5	35.3	26.5
4′	29.6–29.9	29.4–29.9	29.2–30.0	29.3–29.7	27.0	29.5–30.2
5′	29.6–29.9	29.4–29.9	29.2–30.0	29.3–29.7	29.3–29.6	29.5–30.2
6′	29.6–29.9	29.4–29.9	29.2–30.0	134.1	29.3–29.6	29.5–30.2
7′	29.6–29.9	29.4–29.9	29.2–30.0	134.1	29.3–29.6	29.5–30.2
8′	29.6–29.9	29.4–29.9	29.2–30.0	29.3–29.7	22.5	29.5–30.2
9′	29.6–29.9	29.4–29.9	29.2–30.0	132.5	26.0	29.5–30.2
10′	29.6–29.9	29.4–29.9	29.2–30.0	132.5	132.7	29.5–30.2
11′	29.6–29.9	29.4–29.9	29.2–30.0	29.3–29.7	132.7	29.5–30.2
12′	29.6–29.9	29.4–29.9	29.2–30.0	131.3	26.0	29.5–30.2
13′	29.6–29.9	29.4–29.9	29.2–30.0	131.3	29.3–29.6	29.5–30.2
14′	29.6–29.9	31.9	31.8	29.3–29.7	29.3–29.6	32.0
15′	29.6–29.9	22.8	13.9	129.0	29.3–29.6	22.6
16′	29.6–29.9	14.1	-	126.7	31.8	14.1
17′	22.9	-	-	29.3–29.7	13.9	-
18′	14.2	-	--	29.3–29.7	-	-
19′	-	-	-	31.6	-	-
20′	-	-	-	22.6	-	-
21′	-	-	-	14.0	-	-

*: ^13^C NMR spectra using deuterated pyridine (C_5_D_5_N) as a solvent; #: ^13^C NMR spectra using deuterated chloroform (CDCl_3_) as a solvent.

**Table 4:** Receptor interaction of isolated ceramides, compounds **1**–**17** and hesperidin into the SARS-CoV-2 Spike binding site with hACE2.

Compound	dG Kcal/mole	Receptor	
		Amino Acid/Type of Bonding/Distance (Å)/Binding Energy (Kcal/mole)	
		hACE2	Spike Protein
**Cer-1**	−8.2488	GLU 37/H-donor/3.03/−3.2 ARG 393/H-acceptor/3.37/−0.6	-
**Cer-2**	−8.0390	GLU 37/H-donor/2.85/−7.0	-
**Cer-3**	−8.3111	GLU 37/H-donor/2.88/−2.4	-
**Cer-4**	−9.6464	HIS 34/H-donor/3.45/−1.0 HIS 34/H-acceptor/3.38/−0.7	LYS 403/H-acceptor/2.06/−0.5
**Cer-5**	−8.7077	GLU 37/H-donor/2.71/−3.1 HIS 34/H-pi/3.64/−1.4	GLY 496/H-acceptor/3.04/−1.7 LYS 403/H-acceptor/2.98/−4.9
**Cer-6**	−8.6033	GLU 37/H-donor/2.85/−2.8 GLU 37/H-donor/2.74/−2.8 ARG 393/H-acceptor/2.88/−4.1 HIS 34/H-pi/3.76/−1.4	-
**1**	−6.0580	-	-
**2**	−5.9953	LYS 353/H-acceptor/3.23/−1.8	-
**3**	−5.9901	GLU 37/H-donor/2.94/−6.4	-
**4**	−5.5072	-	-
**5**	−6.0686	HIS 34/H-donor/3.50/−0.6	-
**6**	−6.2414	-	-
**7**	−6.5090	-	ASP 406/H-donor/2.90/−3.7 LYS 403/H-acceptor/3.05/−2.3
**8**	−8.4482	GLU 37/H-donor/2.80/−3.6 HIS 34/H-pi/3.91/−1.3	LYS 403/H-acceptor/3.05/−7.5 ARG 408/H-acceptor/2.99/−2.8 LYS 403/H-acceptor/3.10/−4.7
**9**	−7.7408	GLU 37/H-donor/2.90/−2.2	-
**10**	−5.8042	HIS 34/H-pi/3.72/−1.2	-
**11**	−4.9095	-	-
**12**	−6.5362	-	SER 494/H-donor/2.89/−2.5 LYS 403/H-acceptor/2.93/−3.7 GLY 496/H-acceptor/3.38/−0.9
**13**	−5.8862	HIS 34/H-pi/3.7 3/−1.2	LYS 403/H-acceptor/2.92/−7.0
**14**	−5.4205	HIS 34/H-pi/3.76/−0.7	-
**15**	−6.1762	-	-
**16**	−8.6033	GLU 37/H-donor/2.85/−2.8 GLU 37/H-donor/2.74/−2.8 ARG 393/H-acceptor/2.88/−4.1 HIS 34/H-pi/3.76/−1.4	-
**17**	−4.1495	-	LYS 403/H-acceptor/2.80/−4.2
**Hesperidin**	−6.0117	-	ASP 405/H-donor/2.91/−1.8

**Table 5:** Receptor interaction of isolated ceramides, compounds **1–17**, darunavir and N3 into the SARS-CoV-2 M^pro^.

Compound	dG Kcal/mole	Receptor
		Amino Acid/Type of Bonding/Distance (Å)/Binding Energy (Kcal/mole)
**Cer-1**	−7.4765	HIS 41/H-pi/4.08/−0.7
**Cer-2**	−7.1107	-
**Cer-3**	−7.5529	THR 190/H-donor/2.77/−1.7 GLU 166/H-donor/2.85/−2.4 MET 165/H-donor/3.57/−1.6
**Cer-4**	−7.9390	HIS 41/H-pi/4.38/−0.6
**Cer-5**	−7.4910	-
**Cer-6**	−6.7077	GLY 143/H-acceptor/3.09/−0.7 HIS 41/H-pi/4.74/−0.6
**1**	−5.9111	HIS 163/H-acceptor/2.96/−7.3
**2**	−6.2654	HIS 41/H-pi/4.07/−1.1
**3**	−6.0563	GLU 166/H-donor/3.05/−2.9 HIS 163/H-acceptor/3.06/−5.1
**4**	−6.3289	-
**5**	−6.3581	-
**6**	−5.8555	-
**7**	−6.0696	THR 190/H-donor/2.96/−1.2 GLU 166/H-acceptor/3.10/−1.5 HIS 41/H-pi/3.74/−0.9
**8**	−6.7570	GLN 189/H-donor/3.31/−0.8 GLU 166/H-donor/3.00/−0.7 ASN 142/H-donor/3.37/−0.8 GLU 166/H-acceptor/2.70/−2.3 HIS 163/H-acceptor/3.02/−2.6
**9**	−7.0953	HIS 164/H-donor/3.19/−0.7 CYS 145/H-donor/3.81/−1.1 MET 165/H-donor/3.80/−0.8 MET 165/H-donor/3.57/−1.2 HIS 163/H-acceptor/3.31/−2.9
**10**	−5.1913	GLY 143/H-acceptor/2.94/−2.2
**11**	−5.9792	-
**12**	−6.9449	THR 190/H-donor/2.95/−1.7
**13**	−5.5319	-
**14**	−5.1384	GLY 143/H-acceptor/2.97/−0.4
**15**	−6.7512	PHE 140/H-donor/3.07/−0.8 CYS 145/H-donor/3.78/−1.2 HIS 163/H-acceptor/3.11/−0.7
**16**	−6.7077	GLY 143/H-acceptor/3.09/−0.7 HIS 41/H-pi/4.74/−0.6
**17**	−5.4313	-
**Darunavir**	−6.4501	GLN 189/H-donor/3.16/−2.2 ASN 142/H-donor/3.15/−0.9 GLU 166/H-acceptor/3.01/−4.0 ASN 142/pi-H/4.01/−1.6 GLY 143/pi-H/4.18/−0.6
**N3**	−8.4847	GLN 189/H-donor/2.88/−3.4 CYS 145/H-donor/3.99/−1.5 GLU 166/H-acceptor/3.08/−2.6

**Table 6:** Physicochemical parameters and ADME of isolated ceramides, compounds **1–17**, hesperidin, darunavir and N3.

Compound	TPSA Å^2^	Log *P*_o/w_ (MLOGP)	GI Absorption	BBB Permeant	PAINS Alert
**Cer-1**	89.79	4.98	Low	No	0
**Cer-2**	69.56	5.61	Low	No	0
**Cer-3**	110.02	4.71	Low	No	0
**Cer-4**	110.02	4.67	Low	No	0
**Cer-5**	110.02	3.88	Low	No	0
**Cer-6**	110.02	3.99	Low	No	0
**1**	37.30	3.81	High	Yes	0
**2**	54.37	3.59	High	Yes	0
**3**	37.30	4.29	High	Yes	0
**4**	37.30	4.75	High	No	0
**5**	37.30	4.38	High	Yes	0
**6**	37.30	4.67	High	No	0
**7**	66.76	3.18	High	Yes	0
**8**	145.91	0.89	Low	No	0
**9**	145.91	0.48	Low	No	0
**10**	57.53	4.72	High	Yes	0
**11**	20.23	6.33	Low	No	0
**12**	99.38	3.85	High	No	0
**13**	32.76	5.80	Low	No	0
**14**	40.46	5.7	Low	No	0
**15**	43.70	3.44	High	Yes	0
**16**	110.02	3.99	Low	No	0
**17**	20.23	3.56	Low	No	0
**Hesperidin**	234.29	−3.04	Low	No	0
**Darunavir**	148.80	1.18	Low	No	0
**N3**	197.83	0.38	Low	No	0

## Data Availability

The datasets used in the current study are available from the corresponding author upon reasonable request.

## Supplementary Materials

The following data are available online at https://www.mdpi.com/article/10.3390/metabo11120816/s1, Figures S1 and S2: LC-ESI-HRMS chromatogram of crude extract of *Ulva lactuca* (positive and negative modes), Figure S3: ESI–HRMS chromatogram of compound **1**, Figures S4–S6: ^1^H NMR and ^13^C NMR spectra of compound **1**, Figure S7: GC–MS analysis of fatty acid methyl ester of compound **1**, Figure S8: ESI–HRMS chromatogram of compound **3**, Figures S9 and S10: ^1^H NMR and ^13^C NMR spectra of compound **3**, Figure S11: GC–MS analysis of fatty acid methyl ester of compound **3**, Figure S12: ESI–HRMS chromatogram of compound **4**, Figures S13–S15: ^1^H NMR and ^13^C NMR spectra of compound **4**, Figure S16: GC–MS analysis of fatty acid methyl ester of compound **4**, Figure S17: ESI–HRMS chromatogram of compound **5**, Figures S18 and S19: ^1^H NMR and ^13^C NMR spectra of compound **5**, Figure S20: GC–MS analysis of fatty acid methyl ester of compound **5**.
