# Supplementary material for: Chemical Composition of the Red Sea Green Algae Ulva lactuca: Isolation and In Silico Studies of New Anti-COVID-19 Ceramides
Source: Metabolites. 2021 Nov 29;11(12):816. doi: 10.3390/metabo11120816 (PMC8707969; doi:10.3390/metabo11120816)
Supplement: Supplementary file 1 [file metabolites-11-00816-s001.zip › metabolites-1450619-supplementary.pdf]

Chemical Composition of the Red Sea Green Algae *Ulva lactuca*: Isolation and In Silico Studies of New Anti COVID-19 Ceramides

Additional Experimental Detail

1. **Figure S1.** Chromatogram of LC-ESI-HRMS analysis of crude extract of *Ulva lactuca* (positive mode)
2. **Figure S2.** Chromatogram of LC-ESI-HRMS analysis of crude extract of *Ulva lactuca* (negative mode)
3. **Figure S3.** Chromatogram of ESI-HRMS of compound 1
4. **Figure S4.** ^{1}H NMR spectrum of compound 1
5. **Figure S5.** ^{13}C NMR spectrum of compound 1
6. **Figure S6.** Expansion ^{13}C NMR spectrum of compound 1
7. **Figure S7.** GC-MS analysis of fatty acid methyl ester of compound 1
8. **Figure S8.** Chromatogram of ESI-HRMS of compound 3
9. **Figure S9.** ^{1}H NMR spectrum of compound 3
10. **Figure S10.** ^{13}C NMR spectrum of compound 3
11. **Figure S11.** GC-MS analysis of fatty acid methyl ester of compound 3
12. **Figure S12.** Chromatogram of ESI-HRMS of compound 4
13. **Figure S13.** ^{1}H NMR spectrum of compound 4
14. **Figure S14.** ^{13}C NMR spectrum of compound 4
15. **Figure S15.** Expansion ^{13}C NMR spectral data of compound 4
16. **Figure S16.** GC-MS analysis of fatty acid methyl ester of compound 4
17. **Figure S17.** Chromatogram of ESI-HRMS of compound 5
18. **Figure S18.** ^{1}H NMR spectrum of compound 5
19. **Figure S19.** ^{13}C NMR spectrum of compound 5
20. **Figure S20.** GC-MS analysis of fatty acid methyl ester of compound 5

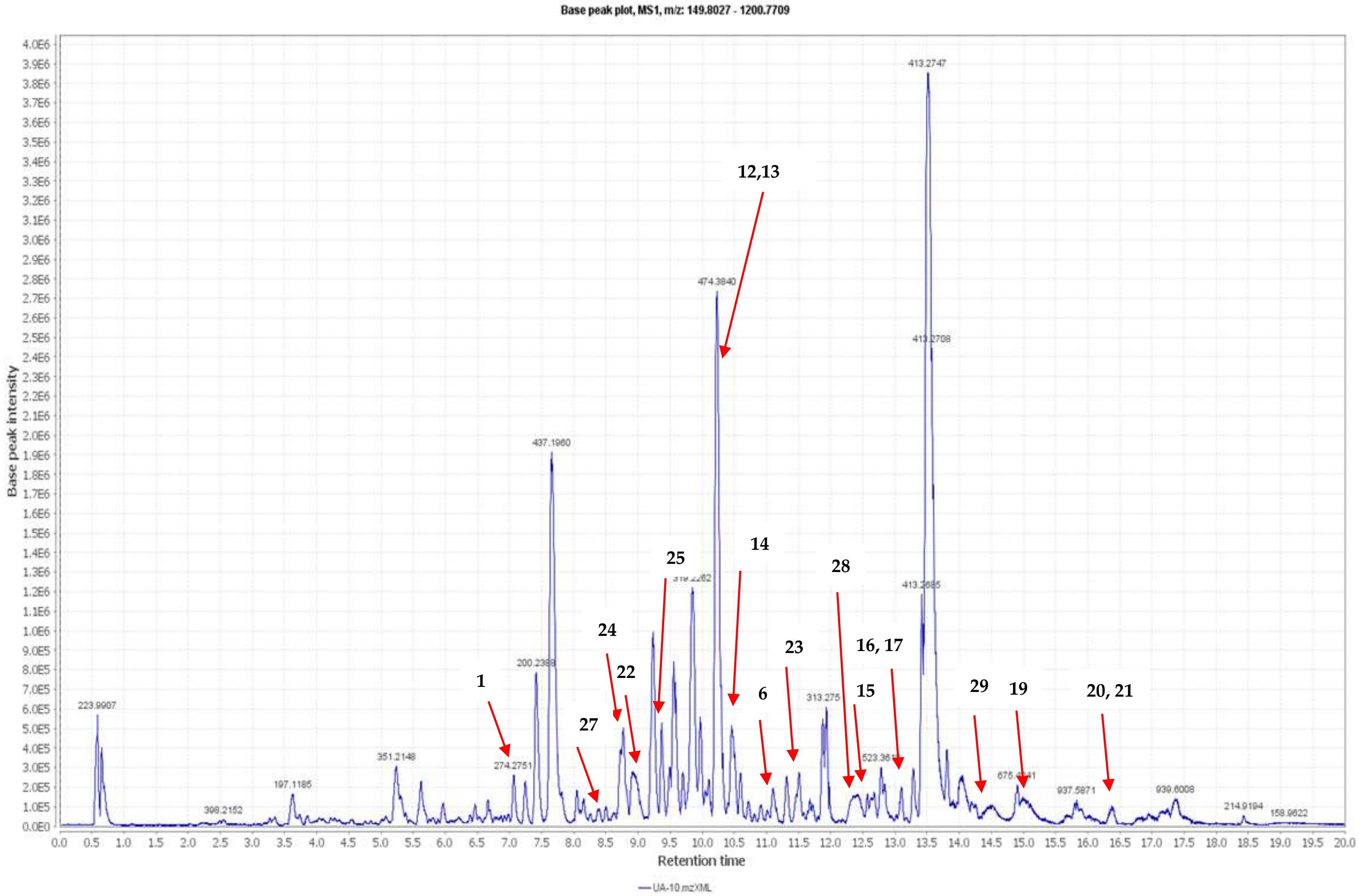

Figure S1. Chromatogram of LC-ESI-HRMS analysis of crude extract of *Ulva lactuca*. (positive mode).

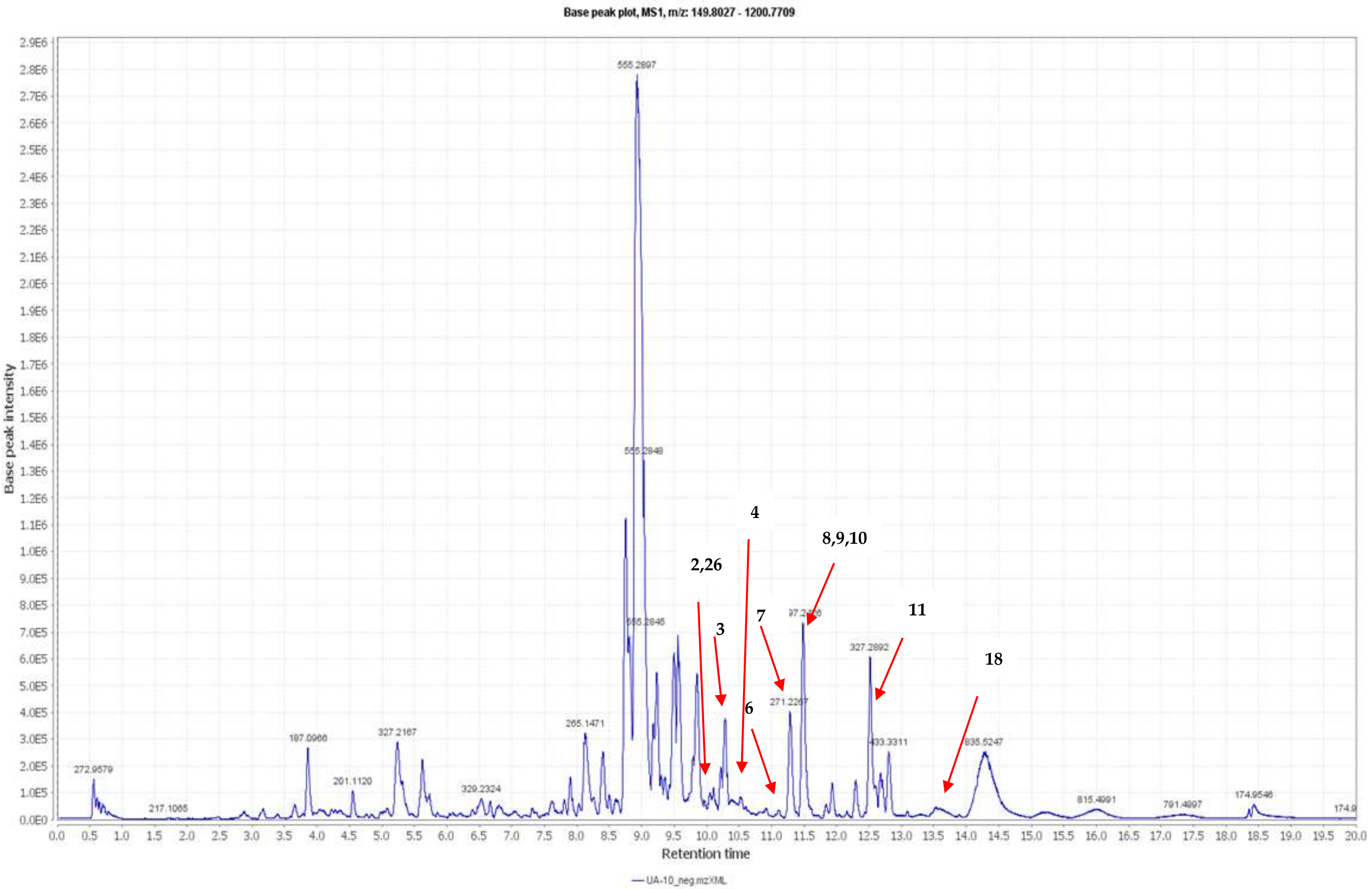

Figure S2. Chromatogram of LC-ESI-HRMS analysis of crude extract of *Ulva lactuca* (negative mode).

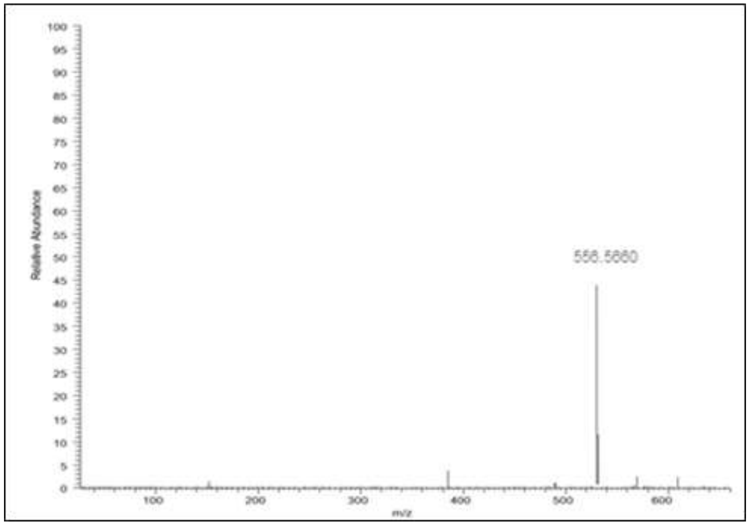

Figure S3. Chromatogram of ESI-HRMS of compound **1**.

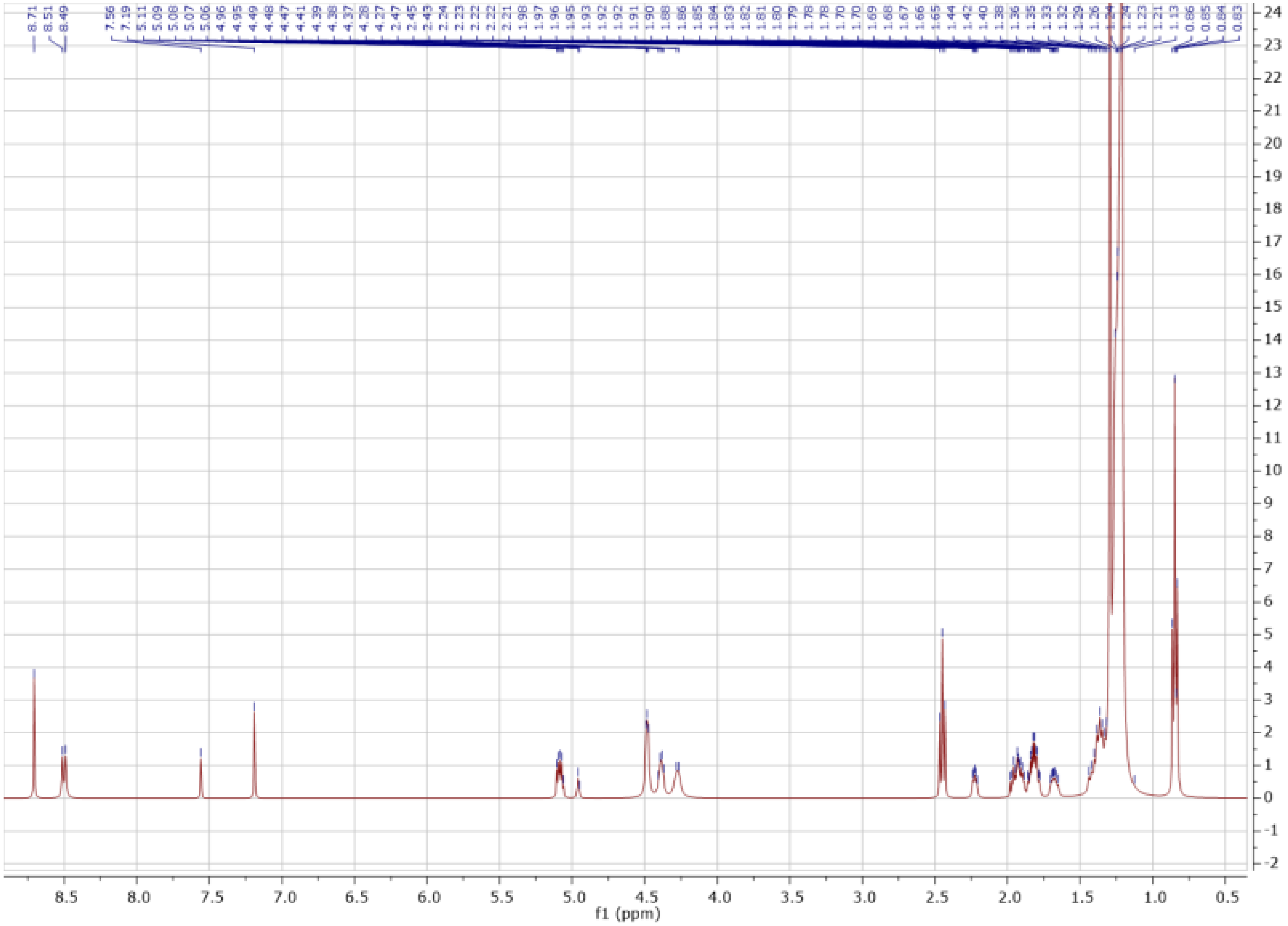

Figure S4. ^{1}H NMR spectrum of compound **1**.

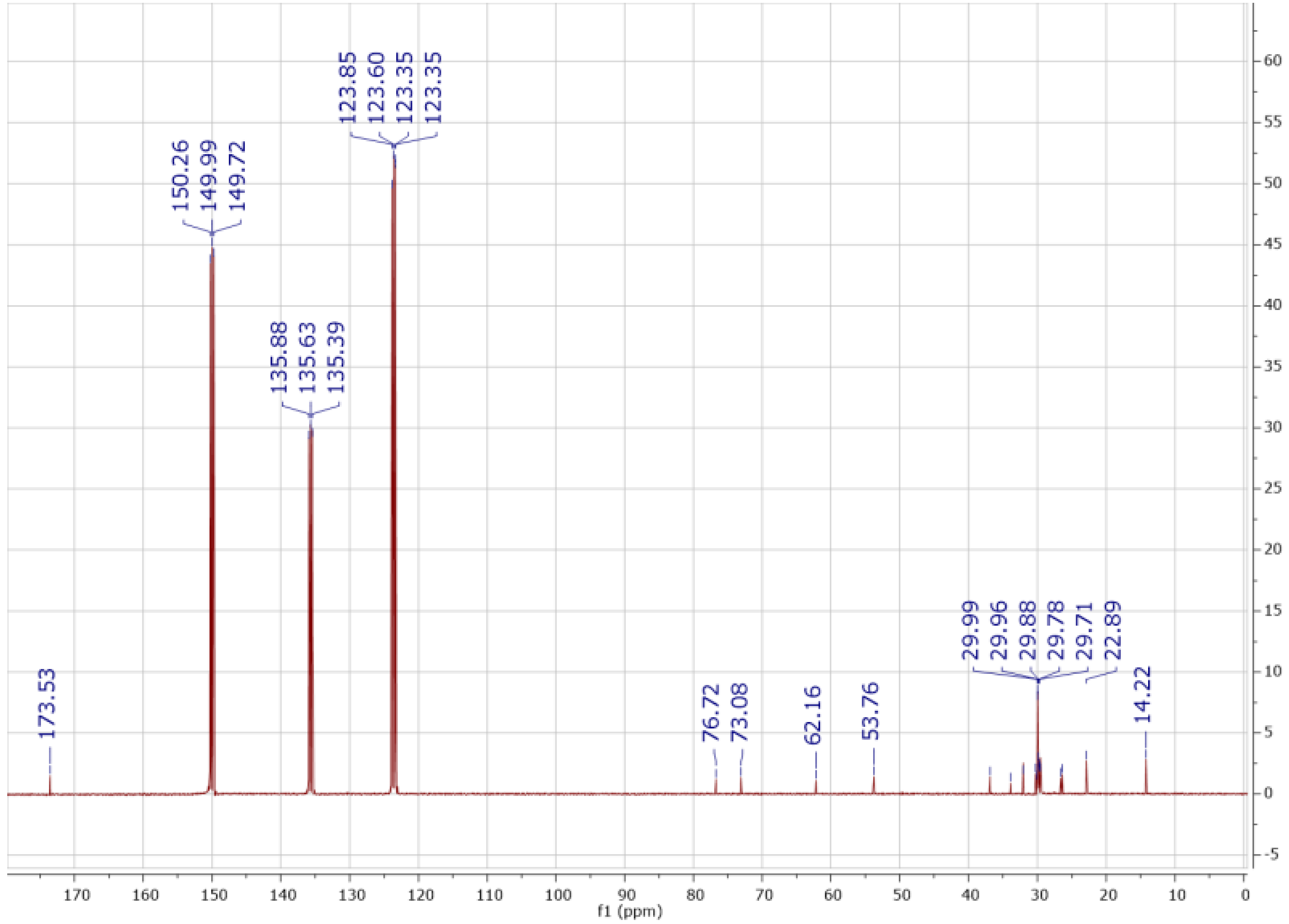

Figure S5. ^{13}C NMR spectrum of compound **1**.

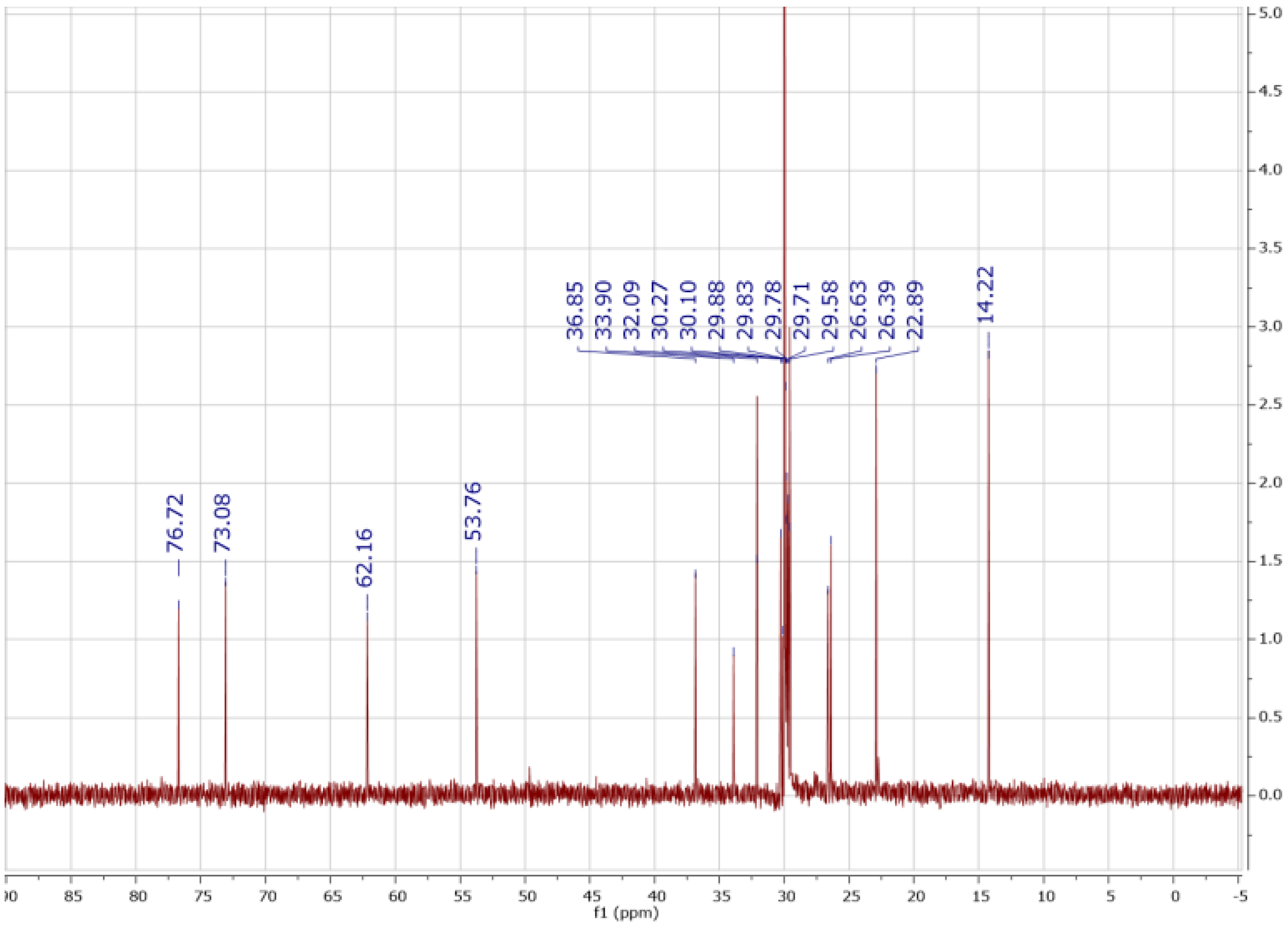

Figure S6. Expansion of ^{13}C NMR spectrum of compound **1**.

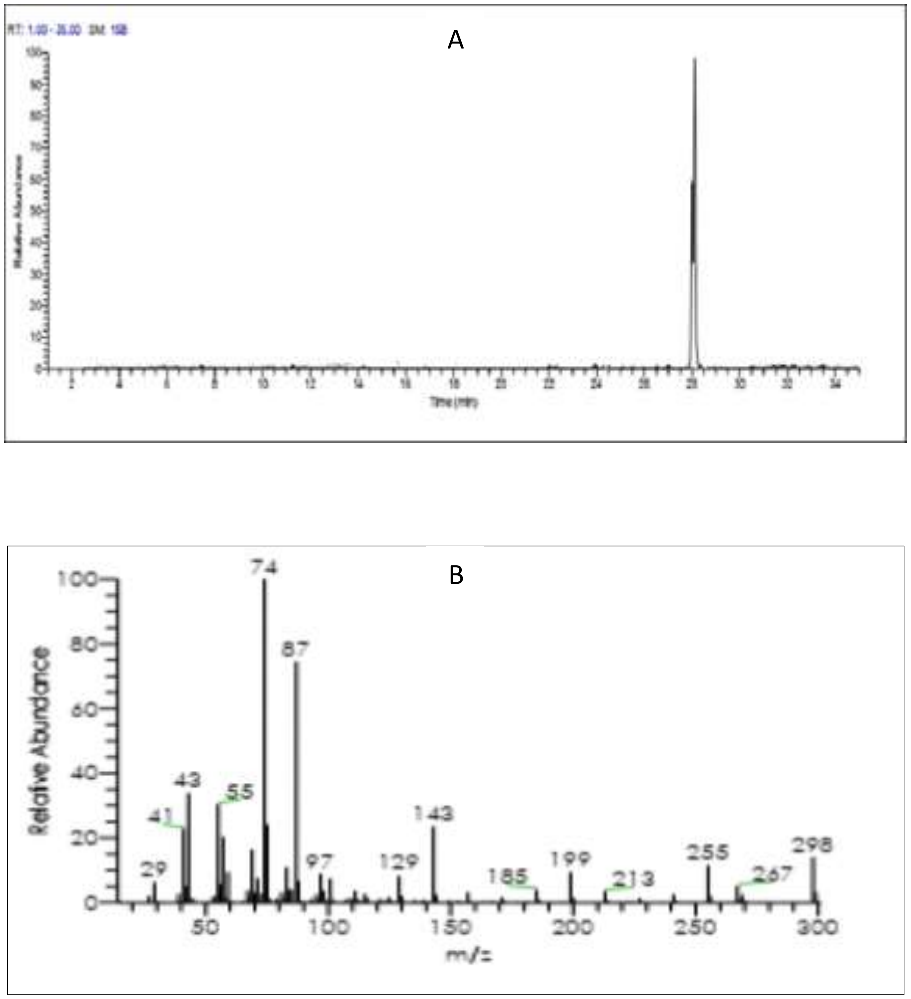

Figure S7. A. GC-MS chromatogram and **B.** Mass spectrum of fatty acid methyl ester of compound **1**.

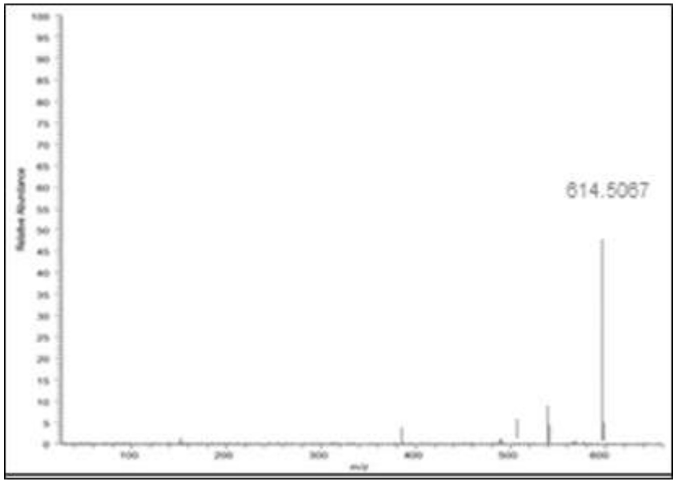

Figure S8. Chromatogram of ESI-HRMS of compound **3**.

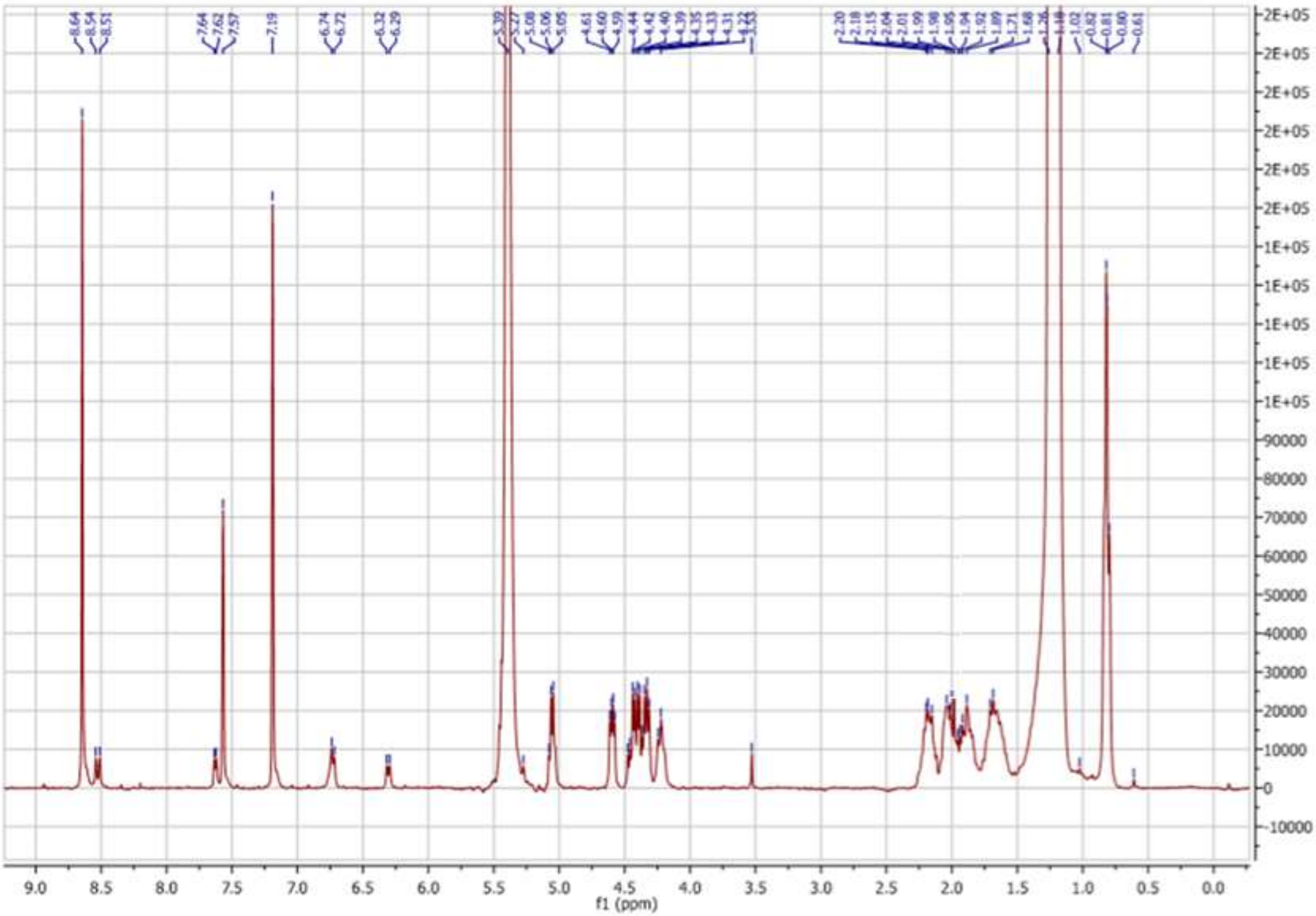

Figure S9. ¹H NMR spectrum of compound **3.**

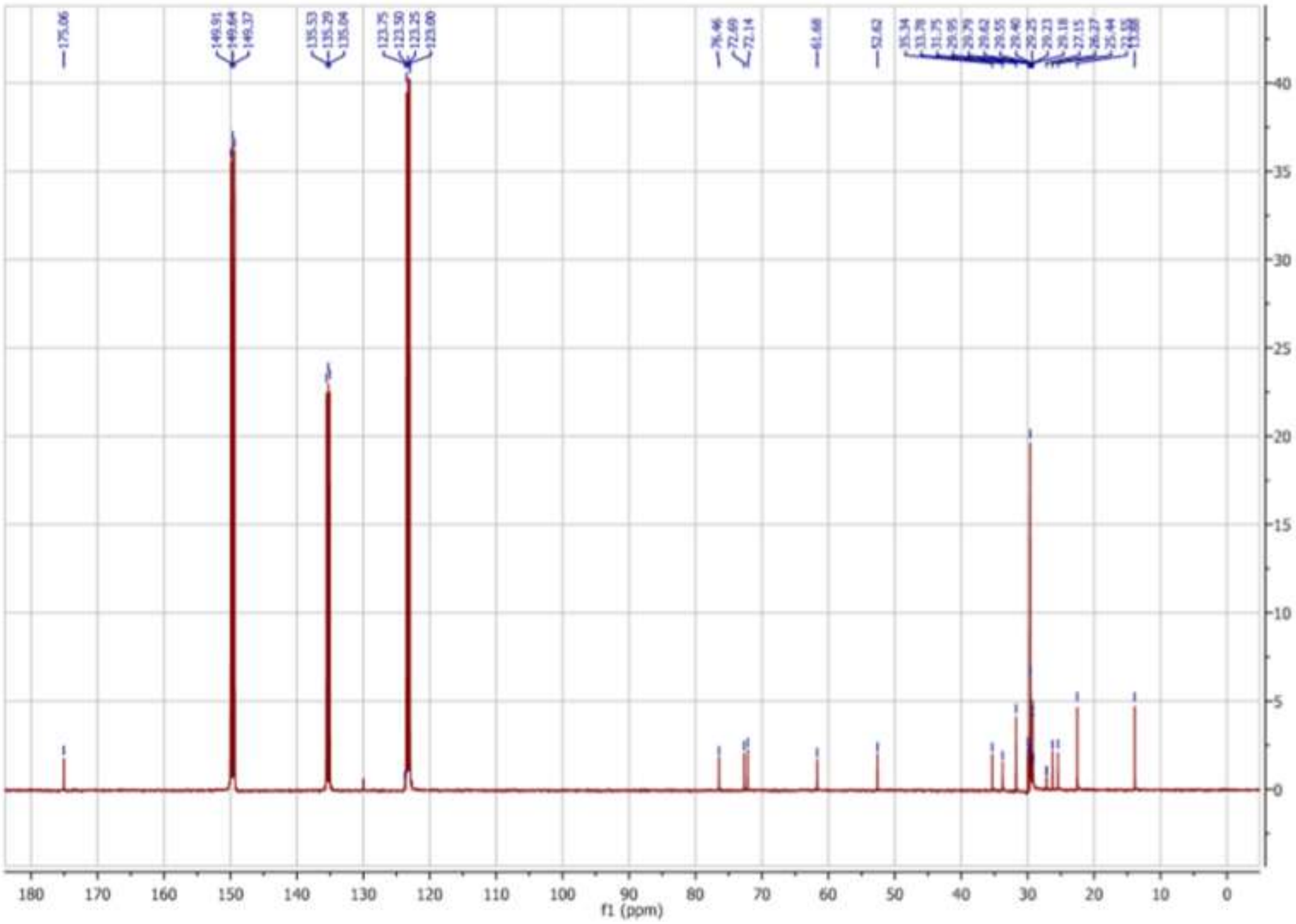

Figure S10. ^{13}C NMR spectrum of compound **3**.

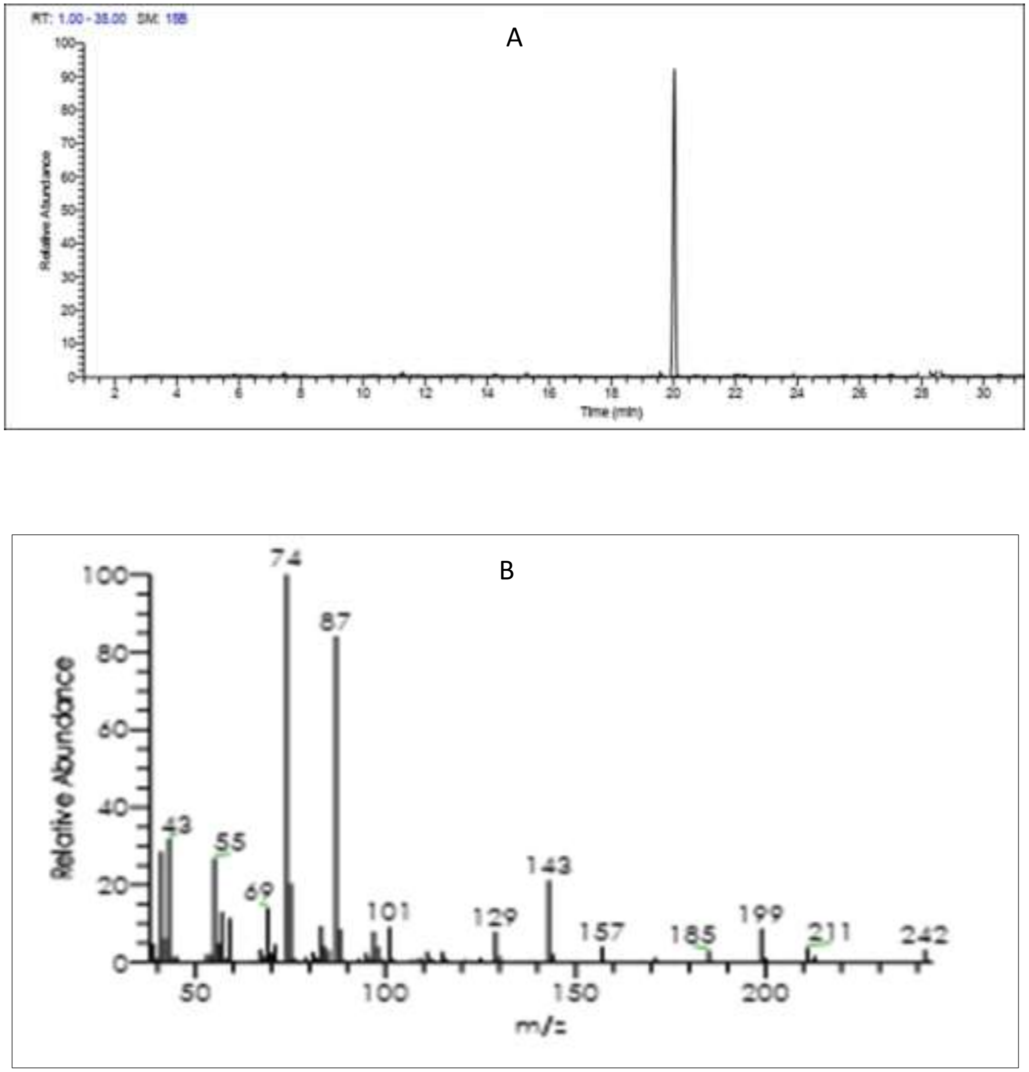

Figure S11. A. GC-MS chromatogram and **B.** Mass spectrum of fatty acid methyl ester of compound **3**.

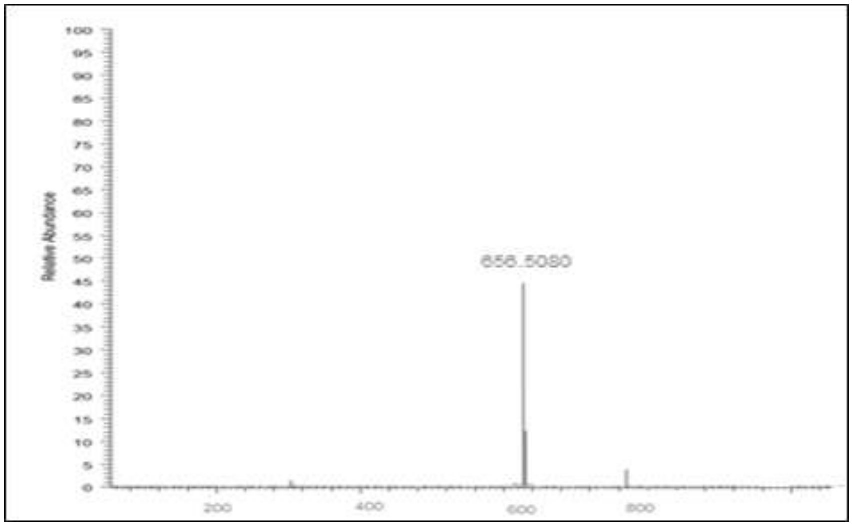

Figure S12. Chromatogram of ESI-HRMS of compound **4**.

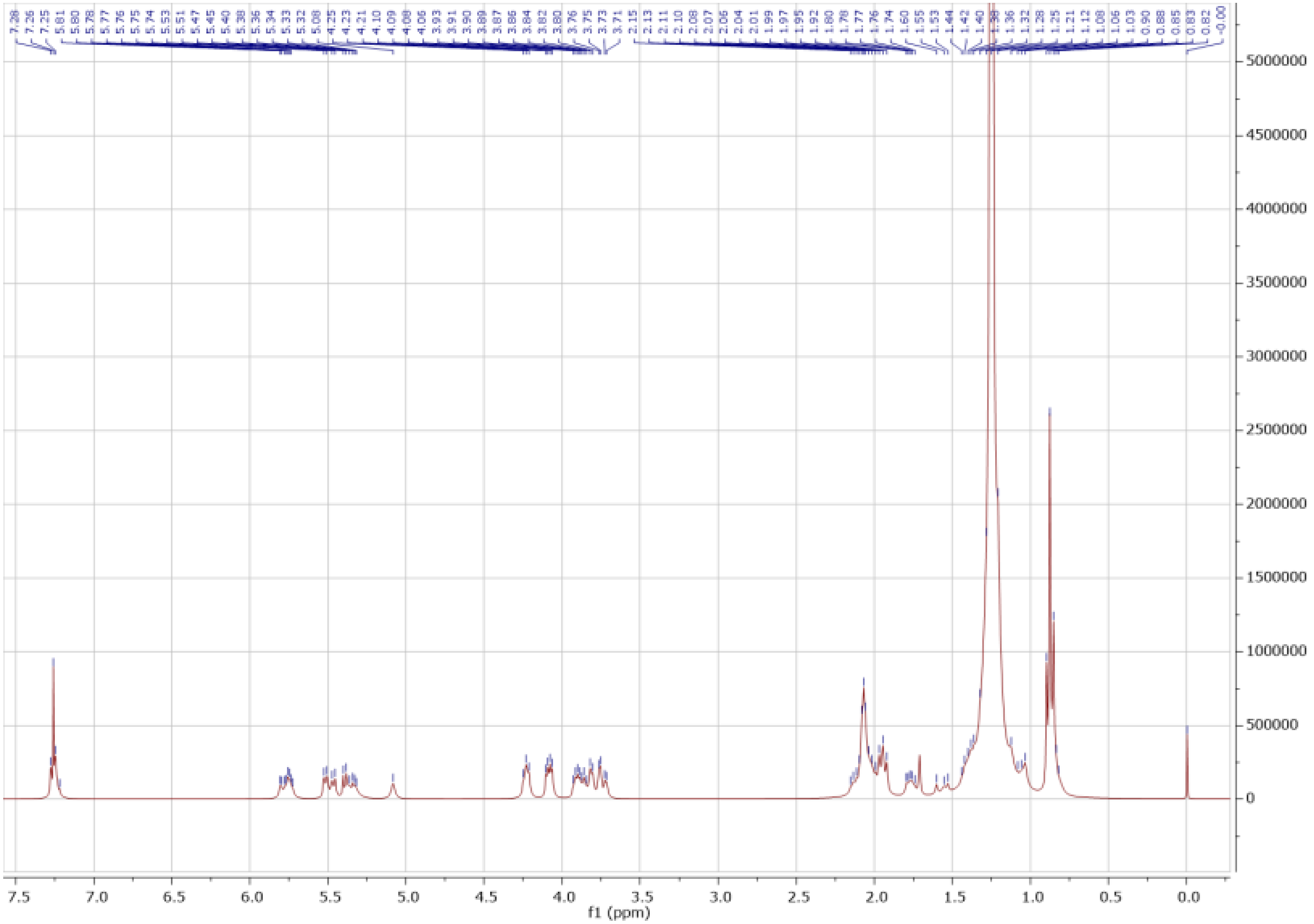

Figure S13. ^{1}H NMR spectrum of compound **4.**

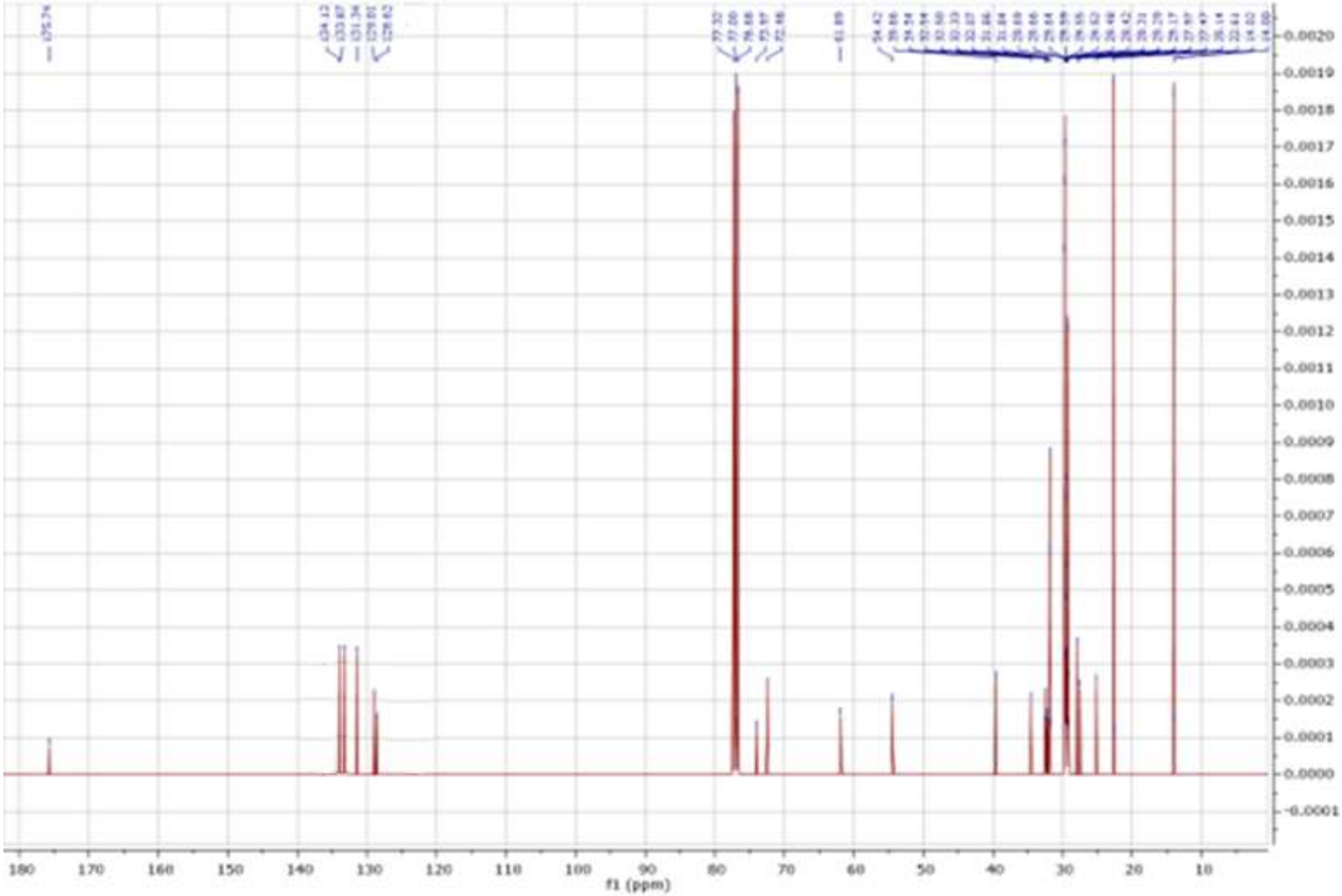

Figure S14. ^{13}C NMR spectrum of compound **4**.

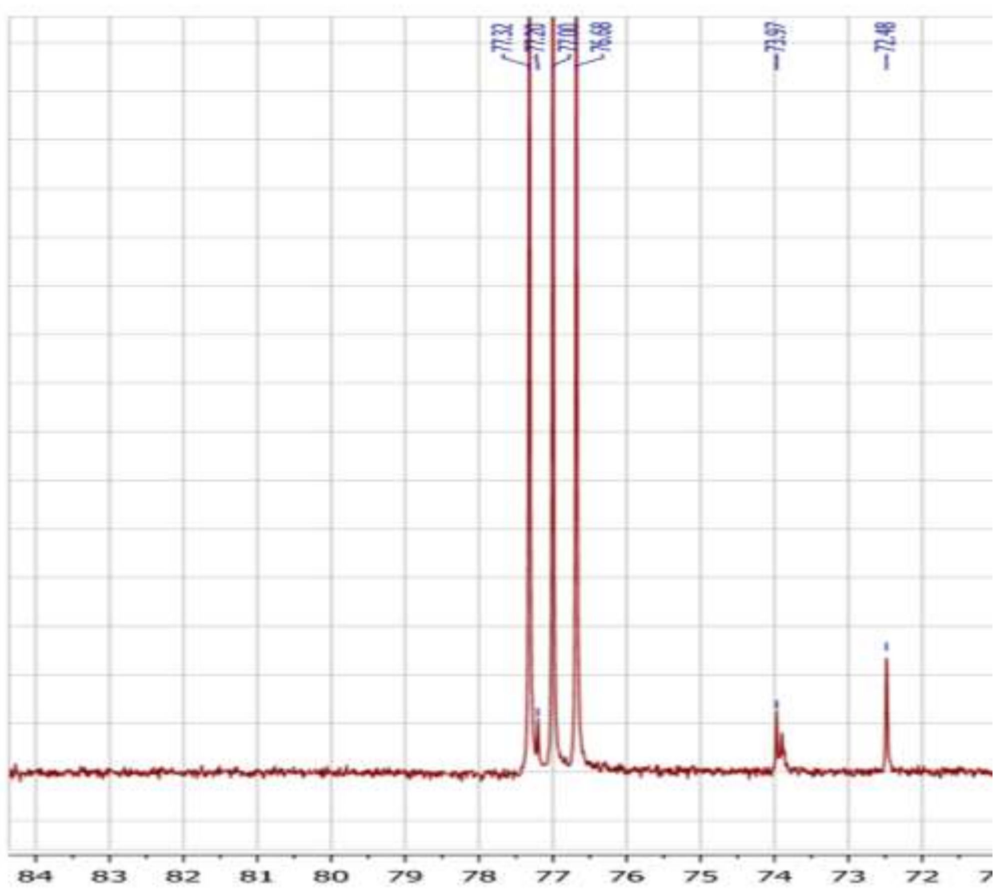

Figure S15. Expansion of ^{13}C NMR spectrum of compound **4**.

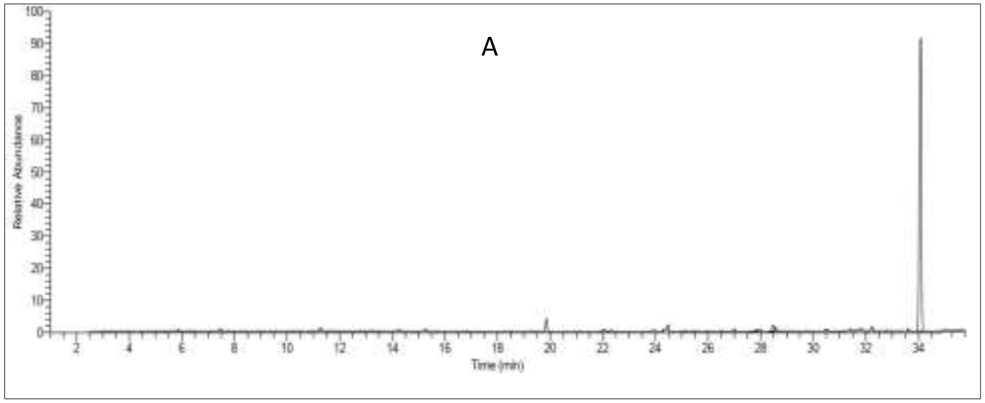

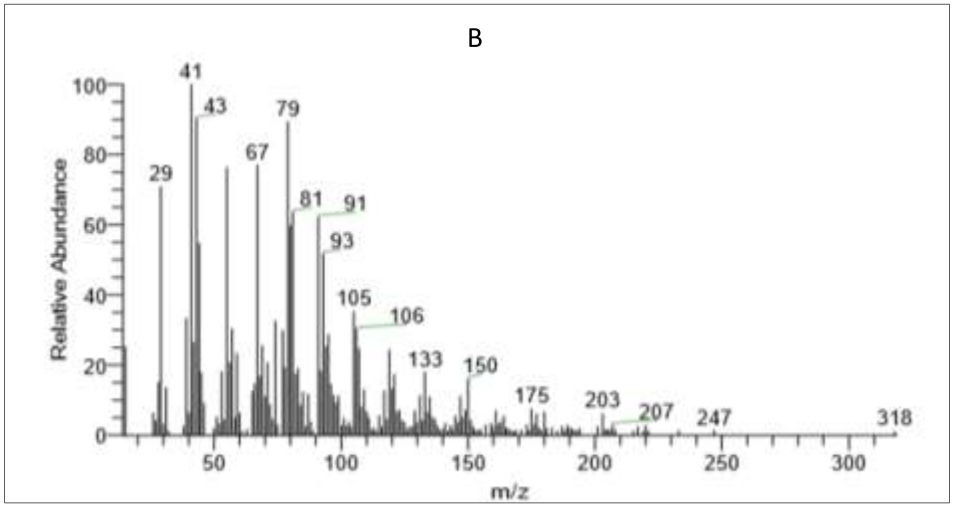

Figure S16. A. GC-MS chromatogram and **B.** Mass spectrum of fatty acid methyl ester of compound **4**

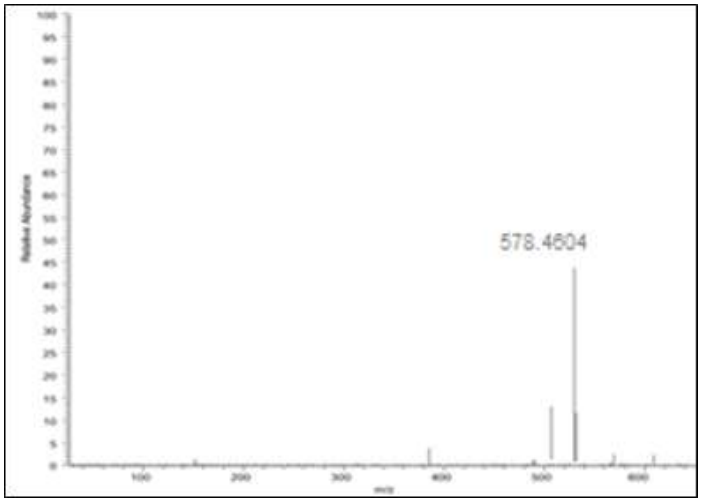

Figure S17. Chromatogram of ESI-HRMS of compound **5**.

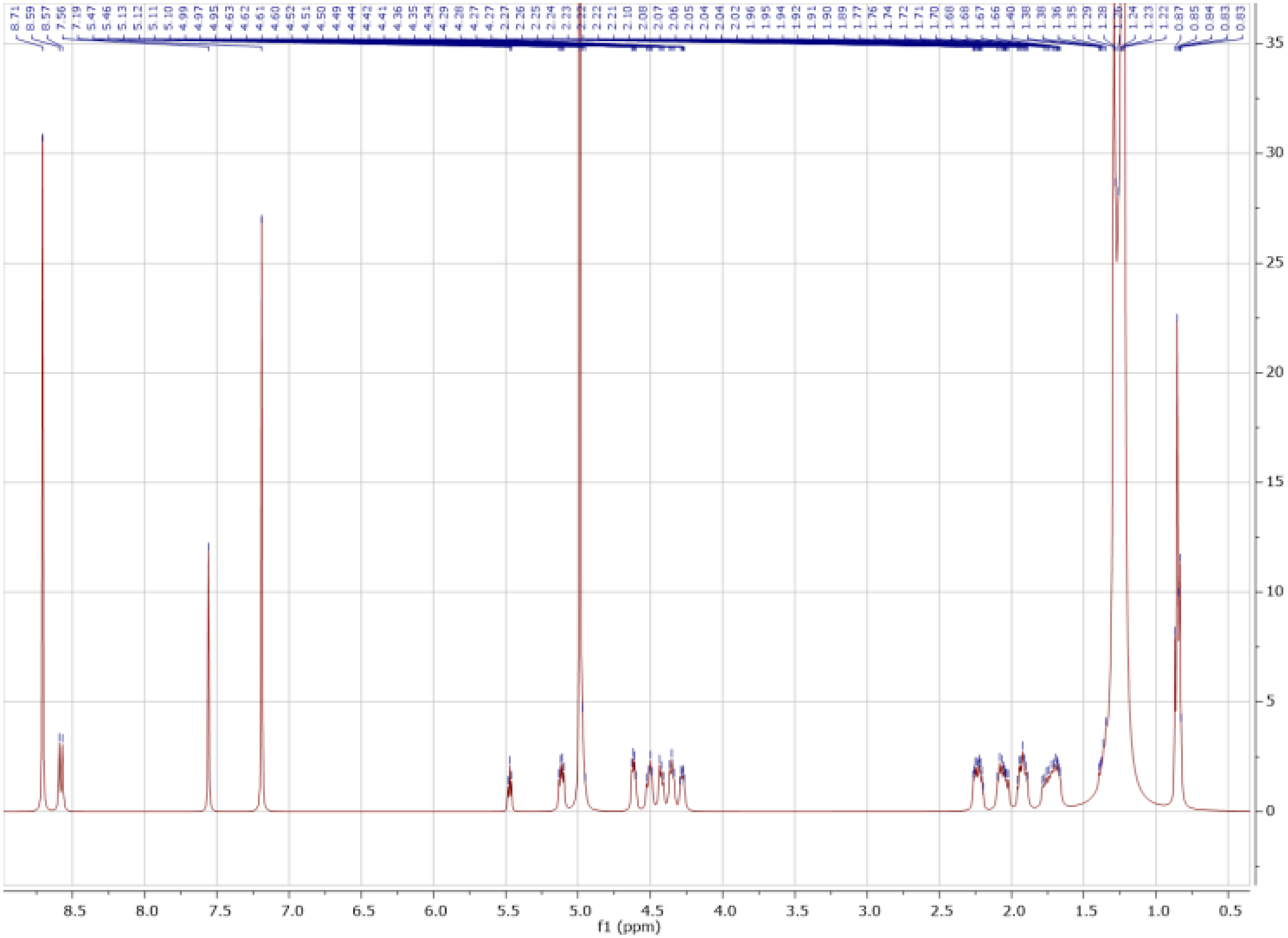

Figure S18. ^{1}H NMR spectrum of compound **5.**

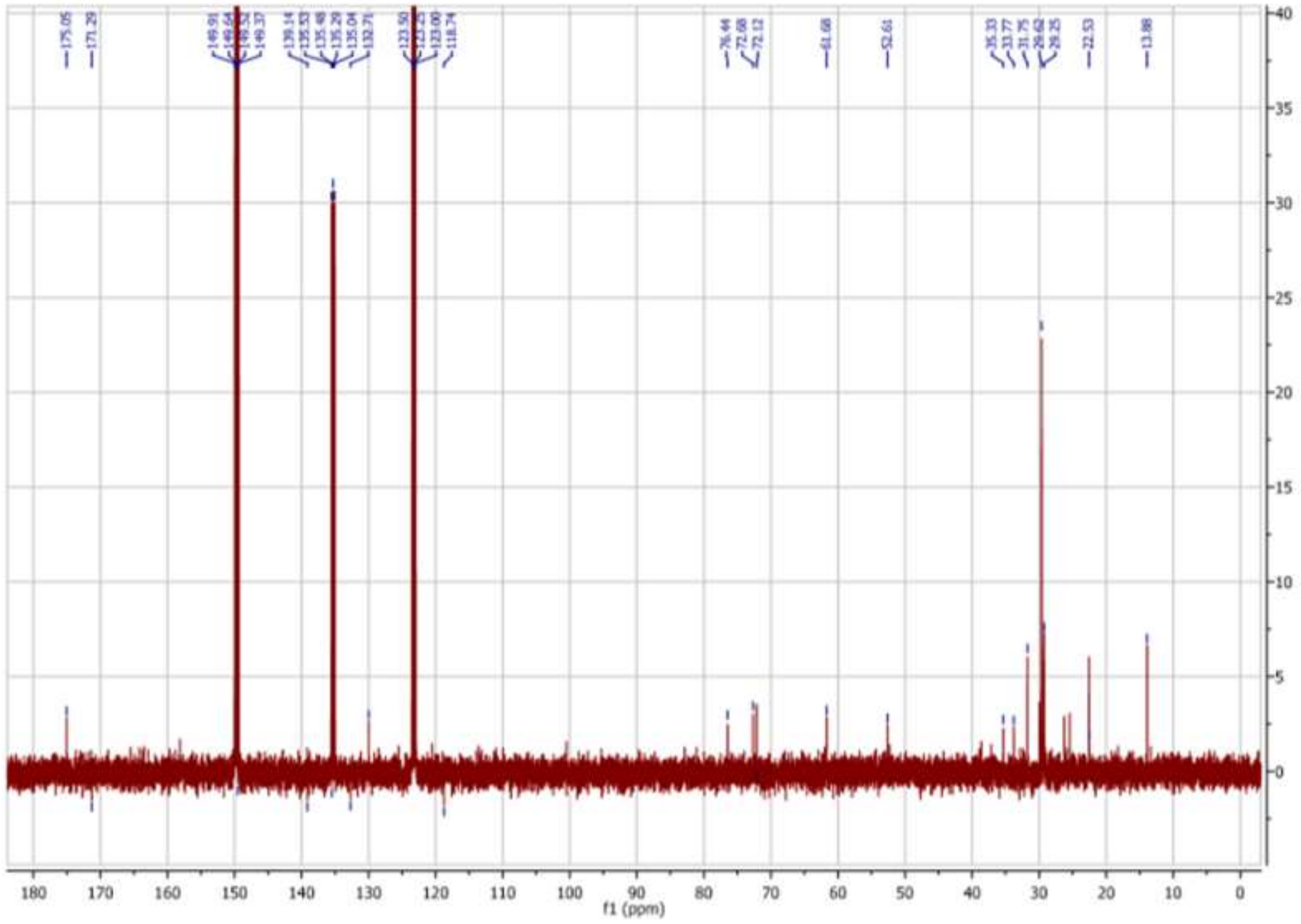

Figure S19. ^{13}C NMR spectrum of compound **5**.

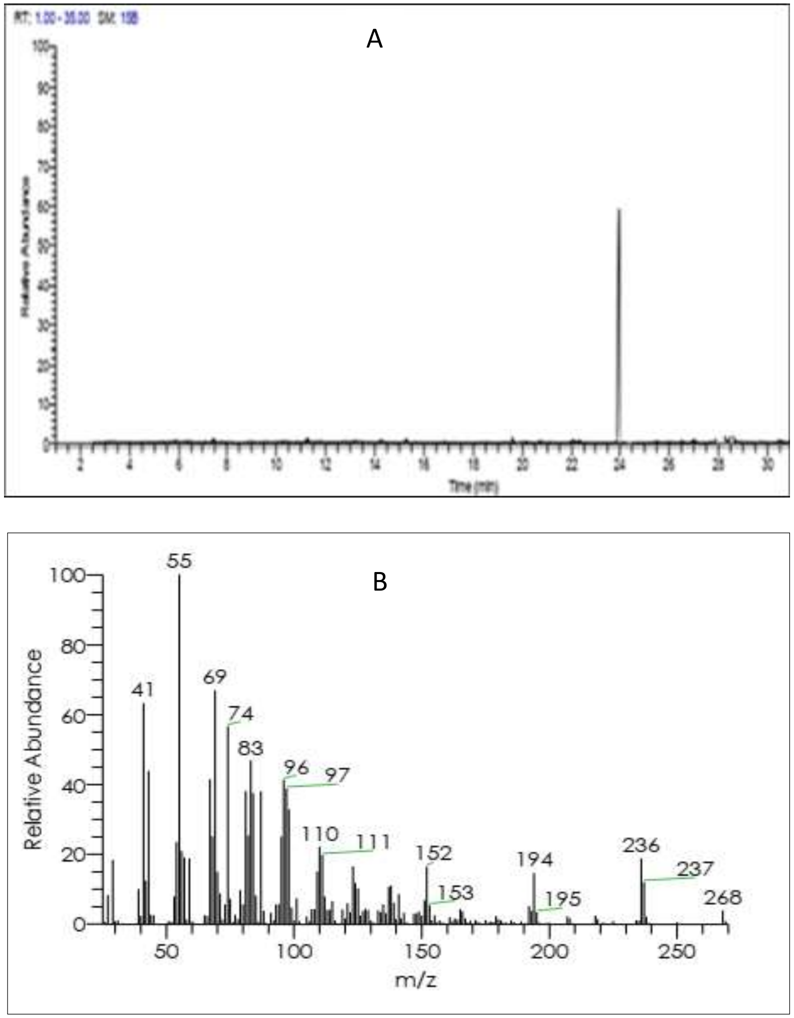

Figure S20. A. GC-MS chromatogram and **B.** Mass spectrum of fatty acid methyl ester of compound **5**.
